# A Crosstalk between Diet, Microbiome and microRNA in Epigenetic Regulation of Colorectal Cancer

**DOI:** 10.3390/nu13072428

**Published:** 2021-07-15

**Authors:** Małgorzata Guz, Witold Jeleniewicz, Anna Malm, Izabela Korona-Glowniak

**Affiliations:** 1 Department of Biochemistry and Molecular Biology, Medical University of Lublin, 20-093 Lublin, Poland; witoldjeleniewicz@umlub.pl; 2 Department of Pharmaceutical Microbiology, Medical University of Lublin, 20-093 Lublin, Poland; annamalm@umlub.pl (A.M.); iza.glowniak@umlub.pl (I.K.-G.)

**Keywords:** nutrition, diet, gut microbiota, gut microbiota-derived metabolites, miRNAs, epigenetic, colorectal cancer

## Abstract

A still growing interest between human nutrition in relation to health and disease states can be observed. Dietary components shape the composition of microbiota colonizing our gastrointestinal tract which play a vital role in maintaining human health. There is a strong evidence that diet, gut microbiota and their metabolites significantly influence our epigenome, particularly through the modulation of microRNAs. These group of small non-coding RNAs maintain cellular homeostasis, however any changes leading to impaired expression of miRNAs contribute to the development of different pathologies, including neoplastic diseases. Imbalance of intestinal microbiota due to diet is primary associated with the development of colorectal cancer as well as other types of cancers. In the present work we summarize current knowledge with particular emphasis on diet-microbiota-miRNAs axis and its relation to the development of colorectal cancer.

## 1. Introduction

**Figure 1:**
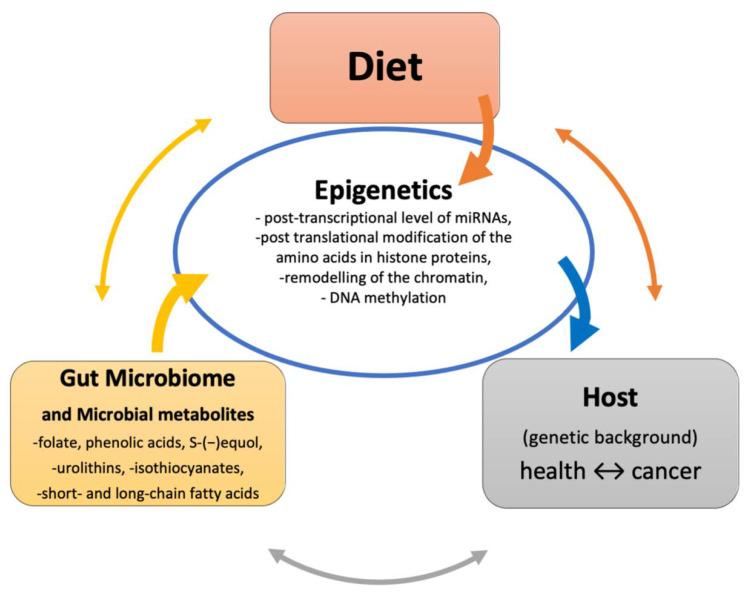
Epigenetic influence through metabolite production by the gut microbiome and diet.

The definition of epigenetics evolved over the centuries. The concept originates in the theory of Aristotle (384–322 BC) who thought that “organs developed in a progressive manner from an originally, undifferentiated homogenous material” [1]. Only in the 17th century the term epigenesis was mentioned by William Harvey as “addition of parts budding out from one another”. Subsequently, in 1940s the term of epigenetics was introduced by the British developmental biologist Conrad Hal Waddington as “inherited changes in phenotype without changes in genotype” [2,3]. At present, epigenetics is defined as a study of reversible and heritable changes in gene expression that modify gene activity and phenotype without any alteration in the DNA sequence. Cooperation and balance between epigenetic changes such as DNA methylation, histones modifications, chromatin organization and regulation of gene expression by RNA interference (RNAi) is required for normal development and homeostasis of multicellular organisms [4]. Particular role in epigenetic regulation seems to be related to micro RNAs (miRNAs), because they not only influence gene expression in epigenetic manner, but can also interact with other epigenetic mechanisms creating important epigenetic link which, when deregulated, can lead to various diseases such as cancer [5]. Each type of epigenetic modification is a dynamic and reversible process which may be affected by genetic and environmental factors such as diet, antibiotics, stress, toxins and microbial metabolites [6]. Microbial bioactive compounds such as short chain fatty acids (SCFAs), choline metabolites and lipids play a fundamental role in triggering host epigenome locally in the gut, but also distally in the liver, heart and central nervous system [7]. Any deregulation of epigenome affects molecular pathways controlling diverse cellular processes what may lead to cancer development and progression [8] (Figure 1).

Diet and dietary habits, influenced by geographical, religious, ethics and cultural aspects, have a vital impact on quality of life, health and lifespan. There have been several approaches to food regimen in order to establish medical and functional diets to prevent and treat health problems. Doll and Peto estimated that 90% of stomach and large bowel cancer-related deaths could be attributed to dietary factors [9]. Hence, the dietary patterns seem to be potentially effective for triggering or preventing cancer directly or indirectly via modulating gut microbial composition and microbial metabolism. Bacteria and their metabolites can influence different signaling pathways, cause DNA double-strand breaks, promote apoptosis, alter cell differentiation, induce inflammation and help to maintain the homeostasis of the body. The gut microbiota is one of the main “organs” affected by dietary intake and has emerged as an important effector which creates association between diet and cancer [10,11]. The microbiome is commonly defined as the collective genomic content of all microbes living in a specific environment. The microbiota comprises all living microorganisms (by type), including mainly bacteria, archaea, fungi, viruses and small protists, forming the microbiome [12]. This endogenic microbiota of gastrointestinal tract is not fully understood so far [13]. Recently, molecular techniques as 16S rRNA sequencing or metagenomics approaches provided greater information about both taxonomy and the whole genome of microbiota (so-called microbiome), unraveling several potential functions of the gut microbiota [14].

Here we focus on the relationship between the dietary metabolism, the gut microbiome and miRNAs expression in cancer, with particular emphasis on colorectal cancer (CRC). 

## 2. Colorectal Cancer—An Overview

The most common gastrointestinal cancer is CRC which is the 3rd in terms of incidence and 2nd in terms of mortality in 2020 worldwide among malignant neoplasms [15]. According to expectations CRC mortality in Europe for 2020 is 15.4/100,000 men and 8.6 in women [16]. In recent years the tendency of increasing cases was reported among adults younger than 50 years in Europe, USA, New Zealand and Australia [17,18,19]. This trend should be monitored, as current guidelines in Europe recommend screening from the age of 50 and possible changes in screening programs should be adopted in the future [17]. CRC incidence and mortality correlate with economic development of countries. Analysis published by Wong et al. in 2020 shows that CRC incidence and mortality are rising rapidly in countries with middle—to high levels of Human Development Index (HDI), however are stable or even decreasing in the majority of developed countries [20]. The most important factor influencing the prediction of survival is the stage of CRC at the time of diagnosis. The 5-year relative survival rate ranges from 90% for diagnosis with localized disease to 14% for patients with distant-stage disease [21].

About 90% of CRCs are sporadic cases caused by somatic mutations and <10% are familial cases of CRCs and include familial adenomatous polyposis (FAP), hereditary nonpolyposis colorectal cancer (HNPCC, Lynch Syndrome), MUTYH-associated polyposis, Peutz-Jaghers Syndrome and serrated polyposis syndrome [22]. In sporadic and familial forms of CRC pathology emerges from the glandular epithelial cells of the large intestine which are characterized by increased rates of proliferation and survival, and give rise to benign adenoma or polyps [23]. Only 10% of all adenomas develop to invasive cancer, however the risk increases with the size of the polyps, then invasive cancers are called adenocarcinomas and account for 96% of all CRCs [23].

Significantly increased risk of developing CRC is observed in patients with chronic inflammation of the gastrointestinal tract [24]. Inflammatory bowel disease (IBD) constitutes of Crohn’s disease and ulcerative colitis, and is one of the highest risk condition for CRC behind FAP and HNPCC [25]. IBD-associated CRC accounts ~2% of all CRCs, but the rate of death in patients with a long-standing chronic inflammation ranges from 10–15% [24]. IBD-associated CRC progresses from low-grade dysplasia in the background of chronic inflammation to high-grade dysplasia and invasive carcinoma [26]. This group of chronic diseases with unknown etiology leads to destruction of normal intestinal architecture [24]. Risk factors which increase IBD-related CRC include disease duration, extent and severity, the presence of inflammatory pseudopolyps, coexistence of primary sclerosing cholangitis and family history of CRC [27]. Since IBD affects digestive system, diet appears to be one of the most important environmental factors that influences the course of the disease. It is known that gut microbiota is formed by diet and microbial diversity play important role in gut and systemic homeostasis. Western diet, which is characterized by high intake of saturated fats and carbohydrates, enhances colitis, delays recovery from gut injury and promotes colon carcinogenesis which is associated with extensive changes in expression of immune-related genes in the colon mucosa [28].

Modifiable risk factors such as smoking, unhealthy diet, high alcohol consumption, overweight, low physical activity, consumption of processed and red meat are the cause of over half of cases and deaths [21]. Numerous studies demonstrate that red meat, processed meat and refined grains are positively associated with the risk of CRC [29]. Tumors accumulate mutations in oncogenes such as *KRAS* and dietary heme intake from red meat shows strong association with tumors that display this defect [30,31]. High level of heme iron increases the formation of endogenous N-nitroso-compounds with mutagenic and carcinogenic potential, and reactive oxygen species (ROS) that exhibit cytotoxic effects and promotes cancer-forming processes by damage of DNA [32]. This leads to imbalance in gut microbiota and increases the rate of gut pathogenic bacteria, inflammation and carcinogenesis. Such events may be reduced by consumption of fresh vegetables and fruits, which are rich in antioxidants decreasing DNA damage and negative impact of N-nitrosocompounds, heme iron and ROS [33]. Furthermore, the intake of sweets, soft drinks and snacks leads to obesity, one of the factors predisposing to CRC [34]. Consumption of artificial sweeteners is prevalent and is expected to increase in the near future [35]. One of them is sucralose, which is used in wide variety of foods and beverages all over the world. Despite the fact that sucralose have been considered healthy, independent studies have shown that sucralose promotes gut damage, inflammation and colitis-associated CRC in murine models along with dysbiosis of gut microbiota [36].

The most important molecular pathways underlying a multi-step carcinogenic process involve chromosomal instability (CIN), microsatellite instability (MSI) and CpG island methylator phenotype (CIMP) [37]. MiRNAs are also mentioned among various genetic and epigenetic mechanisms implicated in CRC development [38]. The discovery of the RNAi was a milestone achievement in understanding the mechanism of regulation of gene expression. This process involves small RNAs such as siRNA and miRNA, with a length shorter than 30 bp that controls the expression of protein-coding genes in the various eukaryotic organisms [39]. The function of miRNA is to negatively regulate the expression of genes through binding to the target mRNA resulting in transcriptional repression, mRNA degradation or both [40]. The function of miRNA depends on the degree of miRNA-mRNA complementarity [41]. A fully complementary miRNA-mRNA promotes AGO-mediated mRNA degradation through its slicing by AGO2, but not fully complementary interactions lead to the repression of translation and mRNA decay which is caused by most of the miRNAs in animal cells [42,43]. The development of CRC as transformation of normal epithelial cells to adenomatous polyps and then cancer is a progressive process under control of different molecular pathways: Wnt, MAPK, PI3K/Akt, TGF-ß, JAK/STAT, NF-κB, aberrant cell cycle and apoptosis which are also regulated by miRNAs [37,44]. In cancer, deregulated miRNAs act as oncogenic miRNAs (oncomirs) or tumor suppressors, where oncomirs that inhibit tumor suppressor proteins are overexpressed, and suppressor miRNAs that regulate expression of oncoproteins are downregulated or even deleted [45]. Potentially mimics of suppressor miRNAs or oncomir inhibitors may be used for therapeutic purposes in CRC what was demonstrated in vitro and in vivo [46,47,48,49,50,51]. Examples of recently published oncomirs and suppressor miRNAs together with their direct targets and effects in CRC are presented in Supplementary Materials (Supplementary Tables S1 and S2).

## 3. Gut Microbiome and Colorectal Cancer Development

Human microbiome includes at least 1000 different species of known bacteria with more than 3 million genes involved in many vital functions such as digestion of carbohydrates, competition with pathogens, vitamin synthesis, immune system activity and drug metabolism. 

The close relationships between host and its commensal microorganisms legitimizes the theory of co-evolution of the host and its associated microbiota [52,53]. In the holistic theory, the host and its commensal microbiota appear as one unit (so-called holobiont), that co-evolves as one entity. According to this approach, “pathobiome” state is the disease state of the holobiont which is linked to dysbiosis characterized by low microbial diversity, variability and increase of proinflammatory species. The healthy state of organism is related to eubiosis, that is high diversity, and uniformity of the individual microbiota [54,55]. Microorganisms constantly make decisions about the costs and benefits of expressing virulence versus controlling population growth. When nutrients are available and antagonism is limited, microbial communities function in a stable, low-growth state, sharing nutrients. In opposite, when they are in distress by vicious competition for nutrients (during extreme physiologic stress of the host), microbial communities can destabilize leading to host tissues invasion, upregulation of multiple defense mechanisms such as biofilm formation, and even expressing a toxic offense (type III secretion). Bacteria have developed many mechanisms to sense and respond in a context-dependent manner. With the use of a variety of membrane-based and multi-layered information processing systems they can recognize signals both physiochemical (nutrients, pH, osmolality) and components of the host, cells and environments (cytokines, products of tissue damage) [56]. The healthy composition of human microbiota mainly comprises of Bacteroidetes and Firmicutes, while the remaining 10% is shared between Proteobacteria, Verrucomicrobia and Actinobacteria [57,58,59,60]. Recently, it has been reported that microbiota can be subdivided into different enterotypes, each consisting of particular bacterial genera, but that all appear to have high functional uniformity in spite of host properties, such as age, sex, body mass index and nationality [61,62]. Dysbiosis, appeared as loss of beneficial microorganisms, unlimited growth of potentially harmful microorganisms or reduced overall microbial diversity [63], has been implicated in a wide range of diseases including cancer [64].The precise mechanism which established the contribution of gut microbiome to cancer is not fully known; however, there are several paths by which the gut microbiota may have an important impact on carcinogenesis [65]. Human body is continuously exposed to microorganisms, as well as their toxic metabolites, including potentially oncogenic biologically active compounds metabolized from plant-derived foods [66]. Circulation of toxic metabolites may contribute to cancer onset or progression at locations distant from a particular microbe habitat. Human microbiome studies have revealed significant differences in the relative abundance of certain microbiota in cancer cases compared with control subjects [67]. Moreover, tumor development is associated with migration of microorganisms to other locations in the human body. There is also a well-known association between the gut microbiota and both inflammation and metabolism characterizing a cancer state [66]. Gut dysbiosis promotes inflammation, DNA damage, increased gut permeability and increased estrogen deconjugation which may contribute to tumorigenesis [68,69].

It is now established that some distinctive members of the microbiota are involved in carcinogenesis. *Helicobacter pylori* is the most common example considered by WHO as group I carcinogen, which causes gastric cancer [70]. It was shown that *H. pylori* infection stimulate epithelial-mesenchymal transition (EMT) and migration of gastric epithelial cells through inducing miR-29a-3p expression [71]. Other bacteria such as *Fusobacterium* spp. or an increased abundance of *Escherichia coli* may be involved in the pathogenesis of CRC [72,73]. The majority of the research in this subject has focused on the relationship between gut microbiota and CRC and they have shown that alterations in gut bacteria have been associated with the development of the cancer. It was found by Arthur et al. [74] that colitis-susceptible interleukin-10 (IL-10) deficient mice infected with *E. coli* NC101 had decreased number of microorganisms and microbial diversity indicating a putative difference in the gut microbiota between sick and healthy individuals and more often developed invasive CRC after azoxymethane (AMO) treatment. Moreover, other studies on stool samples in human patients showed that alterations in the gut microbiota are possible to be associated with adenomas and carcinomas, where specific species were more and other were less frequent [75]. For example, CRC risk was associated with decreased bacterial diversity in stool with depletion of Gram-positive, fiber-fermenting clostridia. The increased presence of Gram-negative, proinflammatory genera *Fusobacterium* and *Porphyromonas* suggests a role of inflammation in microbial change in the gut [76]. *F. nucleatum* has been associated with CRC. Numerous studies have shown that anaerobic Gram-negative pathogenic mechanism leading to *Fusobacterium*-related CRC concerns on invasion and adhesion to the epithelial cells by proteins FadA, Fap2 and RadD expressed by bacteria [77]. It was demonstrated that *F. nucleatum* adheres to and invades CRC cells through unique FadA adhesin that stimulates oncogenic and inflammatory responses and proliferation. FadA binds to E-cadherin, activates β-catenin signaling and induces expression of oncogenes, including miRNAs that promote CRC growth [72,78]. In addition, it was shown that FadA modulates Wnt/ß-catenin signaling pathway by activation of Annexin-1 (ANXA1) expression through E-cadherin, and ANXA1 expression is increased only in proliferating CRC cells, but not in non-proliferating and non-cancerous cells [79]. Moreover, recently it was shown that *Fusobacterium* is significantly involved in the proliferation and migration of CRC cells in vitro by direct induction of cyclin-dependent kinase 5 protein and the activation of the Wnt/ß-catenin signaling pathway [80]. FadA may also bind to the vascular endothelial cadherins (VE-cadherin) increasing endothelial cells permeability allowing bacteria to penetrate through endothelium, overcome blood-brain and placental barrier and colonize the distant parts of the human body [81]. The inverse ratio, when the number of *Bifidobacterium* is significantly decreased and *E. coli* increased is a dysbiosis associated with CRC. It was shown that subjects exposed to a high-risk diet but with increasing levels of bifidobacteria over *E. coli* have a significantly lower CRC risk [82]. Microbiota-mediated metabolic activities can contribute to CRC development via production of pro-carcinogenic compounds such as polyamine [83,84,85,86]. The same process is caused by the production of genotoxins and DNA-damaging superoxide radicals by *Enterococcus faecalis* [87].

## 4. Impact of Gut Microbiome and Their Metabolites on Epigenetics

Environmental factors, disease state, age, host genetics and diet are factors shaping gut microbiota and vice versa, intestinal flora plays a role in the pathophysiology of host diseases through affecting gene and protein expression, and also through their specific metabolites [88]. Diet influences the amount and types of bacteria present in the gut [89,90,91]. It was demonstrated that children with diet rich in fiber from fruits, vegetables and whole grains are more likely to have more diverse and numerous microbes than children with diet consisting of more processed food [89]. In turn, Western diet which is characterized by a high content of protein, sugar, saturated fats, refined grains, alcohol, salt, high-fructose corn syrup and low in fiber reduces microbial diversity [92]. Westernized diets considered as pro-inflammatory diets induce microbial dysbiosis, where *Fusobacterium* spp., adherent-invasive *E. coli* and *Enterobacter* spp. are increased. On the other hand, amount of Firmicutes and Bacteroidetes phylla *Faecalibacterium prausnitzii*, *Roseburia hominis*, *Bifidobacterium* spp. and *Prevotella* spp. is decreased [93]. Plant-based diet as anti-inflammatory diet show opposite effects and increase *Bifidobacterium* spp. and *Lactobaccilus* spp. abundance at the expense of *Bacteroides fragilis* and *Clostridium perfringens* [94]. Recent findings suggest that the intake of foxtail millet attenuates colonic inflammation and reduces the risk of AMO and dextran sulfate sodium (DSS)-induced colitis-associated CRC in mouse model. Millet-treatment also increases the abundance of *Bifidobacterium* spp. and Bacteroidales_S-24-7 [95]. Gut flora helps maintain our health by controlling intestinal homeostasis, epithelial function, development of immune system and numerous additional functions of this organ [96]. *Bacteroidetes*, *Firmicutes* and *Actinobacteria*, the main phyla of bacteria inhabiting the intestine, have enzymes that degrade complex dietary substrates [68]. Degradation of macronutrients initiates fermentation, which produces weak acids such as acetate lowering intestinal pH with impact to microbiota composition and host health [68]. Commensal gut microbiota and their metabolites can either promote carcinogenic signaling or serve tumor suppressive functions by digesting and converting dietary nutrients to secondary metabolites [97,98,99]. The regulation of various components of “gut-brain axis”, “gut-liver axis” and gut-lung axis” through the impact on inflammatory cytokines and antimicrobial peptides production can affect the epigenome through metabolism of short-chain fatty acids (SCFAs), vitamin synthesis and nutrient absorption. Only a few microbial metabolites, including folate, phenolic acids, *S*-(−) equol, urolithins, isothiocyanates, short- and long-chain fatty acids have been tested with respect to their potential to influence epigenetic mechanisms [100].

Many studies have reported the importance of increased fiber intake which reduces the exposure of gut epithelial cells to toxic food ingredients by increasing stool bulk which disperse fecal carcinogens and decreases transit time [101]. Food rich in fiber contain a wide range of complex phytochemicals that are metabolized by the gut microbiome to SCFAs, isothiocyanates and polyphenolic derivatives. The interaction of these bioactive nutrients with human gut epithelial cells may modify epigenetic control of gene expression. Healthy diet is associated with sufficient production of SCFAs by microbial cells. A major source of SCFAs in the gut is the anaerobic fermentation of polysaccharides that are indigestible for the host and, therefore, are referred to as microbiota-accessible carbohydrates. The predominant SCFAs derived from bacterial fermentation are acetate, propionate and butyrate (3:1:1 ratio), which can be oxidized to feed the intracellular acetyl-CoA pool and histone acetylation. The fluctuation in the fermentation products is dependent on the fiber source and bacterial pathways dictated by microbial community composition of the intestine [102,103]. The most investigated SCFA in this matter is butyrate synthetized by butyrate-forming bacteria from butyryl-CoA through butyryl-phosphate or using various transferases, with one of them using exogenous acetate [104]. Duncan et al. [105] showed that while *Faecalibacterium prausnitizii* and *Roseburia* spp. derived about 85% of butyrate carbon from external pools of acetate, *Coprococcus* spp. derived only 28%. In addition, different sources of carbohydrates produce different amounts of butyrate ranging from 56% with pectin to 90% for xylan [105]. Regulation of human gene expression can occur through post-translational modification of the amino acids in histone proteins [106]. Bioactive compounds delivered with food and gut microbiota can modify either DNA methylation or histone signatures through a variety of mechanisms. SCFAs have been shown to inhibit cancer cell growth by multiple mechanisms. Butyrate has varied effects on normal colon epithelial cells or tumor cells depending on its concentration and the metabolic state of the cell [107]. Moreover, SCFAs act as histone deacetylase (HDAC) inhibitors in immune cells and adipocytes and can influence levels of histone acetylation [108] and crotonylation [109] and regulate these cells’ transcription through chromatin state [110]. Normal cells near the lumen are exposed to higher levels of butyrate which accumulates in the nucleus and inhibits HDAC. In normal homeostasis, butyrate plays a role in promoting cell turnover of the colonic epithelium. In contrast, metabolism in cancer cells is dominated by aerobic glycolysis (Warburg effect) which uses glucose over butyrate as the growth substrate. Butyrate can then accumulate in the nucleus where it functions as an HDAC inhibitor and inhibits cell proliferation and induces apoptosis [111]. Therefore, a high-fiber diet is likely to enhance circulating levels of SCFAs and histone acetylation due to microbial activity. In mice, microbial colonization has been shown to result in a diet-dependent increase in H3 and H4 acetylation in different tissues, effects that are partially replicated by SCFA supplementation [112]. Colonization of butyrate-producing bacteria in mice with CRC fed a high-fiber diet has tumor-suppressive effects through the inhibition of HDACs by butyrate, which upregulates histone H3 acetylation, activates apoptotic genes and suppresses cancer cell proliferation [113].

In Western Europe more than a half of polyunsaturated fatty acids (PUFAs) are found in plant-derived sources: oils, nuts, grains, predominantly as omega-6-linoleic acid and some omega-3-alpha-linolenic acid, and the rest comes from non-marine animal sources, such as dairy, meat, eggs (linoleic acid, alpha-linolenic acid and arachidonic acid) and from fish as long-chain-omega-3 acids, eicosapentaenoic and docosahexaenoic acids [114]. Recently, it has been proved that in healthy humans, the consumption of omega-3 PUFAs leads to an increased abundance of several butyrate producing bacteria [115]. The anaerobic bacteria from *Roseburia*, *Bifidobacterium* and *Lactobacillus* genera found in the distal gut, metabolize omega-3 polyunsaturated fatty acids from dietary intake to conjugated linolenic acids [116]. Omega-3 PUFAs present anticancer and anti-inflammatory effects and even though they are not produced by bacteria, they may be available due to microbial metabolism what may partially explain some of the individual differences in their effects in cancer. Of note, omega-3 fatty acids formation is affected by the gut microbiota: Wall et al. orally co-administered α-linolenic acid with two strains of *Bifidobacterium breve* to mice and detected elevated levels of eicosapentaenoic acid in the liver and docosahexaenoic acid in the brain [117]. Case-control and experimental studies on a defined dietary intake support the hypothesis of reduced CRC risk by omega-3 PUFAs [118]. Other studies have reported a significant reduction of the tumor size in CRC cells-derived xenograft rodent models supplemented with dietary PUFAs in comparison to control [119]. Methylation is a hallmark of cancer, influences different pathways and is crucial in CRC tumorigenesis [120]. Treatment of CRC cell lines with fish oil and pectin increases apoptosis through increased methylation of Bcl-2 promoter [121]. On the other hand, decreased methylation of tumor-related genes such as PPAR-gamma, E-cadherin, matrix metalloproteinase-2 (MMP2), cyclooxygenase-2 (COX-2) or phosphatase and tensin homolog deletion on chromosome 10 (PTEN) was observed after PUFAs treatment of CRC cells [122]. Expression of demethylases is also influenced by PUFAs in CRC in vitro in a cell-specific manner. Reduced expression of DNMT1 and DNMT3B was caused by docosahexaenoic acid, eicosapentaenoic acid and omega-6-linoleic acid in LS180 and HCT116 cell lines, but DNMT1 expression was enhanced in SW742 and HT29/29 cell lines [120]. Ten-eleven translocation proteins (TETs) hydroxylate 5-methylcytosine to 5-hydroxymetylcytosine, a critical step in the demethylation of DNA is associated with activation of gene transcription and may be associated with catabolism of PUFAs. Increased beta-oxidation of fatty acids also increases concentrations of alpha-ketoglutarate, an intermediate in Kreb’s cycle which induce activity of TETs [122,123].

High-fat diets stimulate production of primary bile acids in the liver that are metabolized to secondary bile acids by gut microbiota [124]. Tightly control of production of bile acids is required, because as an antibacterial agents they control overgrowth of gut bacteria and help to protect intestinal barrier function [125]. Bile acids modulate the gut microbiota composition through antimicrobial effects and the induction of innate immune activity [126]. Wang et al. [127] demonstrated that fecal concentrations of deoxycholic acid were dramatically increased after the cholic acid administration. High deoxycholate acid concentrations inhibit the growth of many intestinal bacteria, including *Clostridium perfringens*, *Bacteroides fragilis*, lactobacilli and bifidobacteria in vitro [127]. Thus, the hypothesis that the complex interactions between bile acids and the intestinal microbiota significantly influence cancer development is reasoned.

Microbial species may regulate the host epigenome by competing with host cells for nutrients. Colonization of choline-consuming strains of *E. coli* in mouse gut was shown to reduce serum levels of methionine-cycle-related metabolites, to induce heritable changes to global DNA methylation and to predispose these animals to high-fat-diet-induced metabolic disorder [128].

The gut microbiota is also involved in the metabolism of folate and B-vitamins. Selected bacteria are able to synthesize folic acid from pteridine precursors and *p*-aminobenzoic acid [98]. Folate deficiency after antibiotic use indicates that colonic folate production can be significant. It is involved in one-carbon metabolism and generation of S-adenosyl methionine (SAM); altered SAM levels influence methylation reactions of DNA and histones. Moreover, the gut microbiota provides a variety of dietary energy metabolites, such as ATP, NAD^+^ and acetyl-CoA, which serve as essential cofactors for many, perhaps most, epigenetic enzymes that regulate DNA methylation, post-translational histone modifications and nucleosome position [129,130]. Human cells depend on a constant supply of biotin from the intestinal microbiota to maintain normal levels of protein biotinylation which is an important epigenetic process that enables the attachment of biotin to histone proteins resulting in gene repression. It also plays a role in DNA repair and chromatin structure [131].

It was reported that bacteria, such as *E. coli*, *Bacteroides thetaiotaomicron*, *Enterococcus* *faecalis*, *Enterococcus faecium*, *Peptostreptococcus* spp. and *Bifidobacterium* spp., isolated from the human gut or stool can convert glucosinolates into isothiocyanates and other derivatives [132]. Controlled feeding studies in humans have shown significant individual differences in the activity or composition of the intestinal bacteria involved in isothiocyanates formation [133]. It was suggested that in vivo exposure to isothiocyanates prevents or reduces tumor growth via the effects on DNA methylation, histone modification and miRNA. An isothiocyanate, sulforaphane, which is an HDAC inhibitor and leads to an increase in general and local histone acetylation, prevents carcinogen or genetically induced CRC in rodent models [134,135].

Dietary polyphenols undergo complex microbial transformations in the colon and both these compounds and their metabolites were identified in systemic circulation. Moreover, they exhibit selective action against different species in the microbial community by either acting as an antibiotic or a prebiotic which may influence human exposure to microbial metabolites of polyphenols [136,137]. Additionally, the microbial metabolites of epigallocatechin-3-gallate (a polyphenol found in green tea), gallic acid and epigallocatechin influence epigenetic gene expression by acting as histone acetyltransferases inhibitors [118,138], although not as strongly as epigallocatechin-3-gallate. Ellagitannins, polyphenols found in fruits (raspberries, strawberries, blackberries) and nuts (walnuts and almonds) have strong antioxidant, radical scavenging, anti-viral, anti-microbial, anti-mutagenic, anti-inflammatory, anti-tumor promoting and immunomodulatory properties [139]. The gut microbiome metabolizes ellagitannins to urolithins with big individual variation in urolithin production [140]. Recent studies suggest that bacteria from the *Clostridium coccoides* group and *Actinobacteria* are involved in the production of urolithins, namely urolithin D, urolithin C and urolithin B that were associated with an increase in amount of *Bifidobacterium* spp. and *Lactobacillus* spp. [139]. Urolithins have a broad spectrum of bioactivities in vitro and in vivo, including antioxidative, anti-inflammatory, anti-estrogenic and anti-proliferative activities [141]. Several studies have addressed the question whether ellagitannins and urolithins target epigenetic mechanisms, with a focus on miRNAs [142]. In vivo urolithin A stimulates expression of miR-10b-5p in CD4^+^ T cells and may be a natural immune-suppressant in patients with IBD whose are at increased risk of development CRC [143]. The effects of urolithins on CRC were confirmed in vitro. Urolithins downregulate miR-224, but upregulate miR-215 with induction of *CDKN1A* gene expression confirming anti-proliferative and cell-cycle blocking effects on CRC cells [142].

The absorption and excretion of minerals such as zinc, selenium, cobalt, iodine, etc. that are important enzymes cofactors participating in epigenetic processes are contributed by gut microbiota. The gut microbiota is also a purveyor of various enzymes such as the methyltransferases, acetyltransferases, deacetylases, Bir A ligase, phosphotransferases, kinases and synthetases [130].

Fasting as abstinence from all solid food for a period of time is a dieting approach with beneficial effects for health [144]. One of the main mechanisms by which fasting induces metabolic improvements is mediated by the gut microbiota [145]. Increasing the levels of *Firmicutes* while decreasing most other phyla and rising the production of SCFAs accordingly in every-other day fasting animals when compared to control animals were observed. Food abstinence decreases the abundance of potentially pathogenic Proteobacteria while increasing *Akkermansia muciniphila* levels [146]. The effect of periodic fasting on the human gut microbiota, SIRTs expression and mitochondrial content in 51 males and females were analysed by Lilja et al. [147]. Periodic fasting triggered a significant switch in metabolism, as indicated by the increase in ß-hydroxybutyrate (BHB) and pyruvate dehydrogenase kinase isoform 4 (PDK4) expression in the capillary blood. MtDNA, SIRT1, SIRT3 and miR-let-7b-5p expression in blood cells were elevated. Following fasting, gut microbiota diversity increased and a statistically significant correlation between SIRT1 gene expression and the abundance of *Prevotella* spp. and *Lactobacillus* spp. was detected. SIRT1, along with histone deacetylation, regulates transcription factors, such as p53 [148], and DNA repair proteins, such as poly ADP-ribose polymerase 1 (PARP1) [149] being a protection from a wide array of metabolic and age-related diseases, such as cancer, cardiovascular and neurodegenerative diseases.

## 5. Diet, Gut Microbiota and Their Metabolites Affect miRNAs Expression in CRC

Many reports present that microbiota affects host miRNAs expression. These miRNAs play a role in physiologic process such as cell proliferation, differentiation and apoptosis, but their deregulation leads to pathologies including cancer [33]. Aberrantly expressed miRNAs influence molecular changes leading to initiation, development and progression of the tumor [33,150]. Development of CRC is the effect of the decline of butyrate-producing bacteria and increasement of detrimental bacterial populations [151]. Butyrate represents anti-CRC effects through regulation of miRNAs. SCFAs content is significantly diminished in plasma of patients with CRC, and this confirms the fact that lower SCFAs levels promotes CRC progression [152]. In vitro butyrate lowers the expression of oncogenic miR-17-92 cluster members, including miR-92a, miR-17, miR-19a/b, miR-20. It was caused by lowering the expression of c-Myc that reduces c-Myc recruitment on miR-17-92a promoter [153]. As transcription factor, c-Myc regulates expression of genes and influences the characteristics of colon cancer stem cells; its role in CRC was confirmed by genetic ablation in mouse models that promotes tumorigenesis [154]. Earlier research results showed that butyrate in CRC cell lines reduces the level of c-Myc at different stages: by suppression of mRNA transcription, acceleration of mRNA degradation and inhibition of mRNA splicing that leads to suppression of transcription of oncogenic pri-miR-17-92, precursor and mature miR-92a [153]. In consequence, expression of cyclin-dependent kinase inhibitor 1C (CDKN1C; p57) is increased which induces apoptosis and inhibits proliferation of CRC in vitro [153]. In CRC p57 is considered as a tumor suppressor, and its downregulated expression is due to hypermethylation of promoter through increased activity of methyltransferase DNMT3a [155].

It was confirmed that butyrate upregulates miR-203 expression and inhibits clone formation, proliferation, invasion of CRC cells and also induces apoptosis by inhibition of neural precursor cell expressed developmentally down-regulated gene 9 (NEDD9)—predictor of poor outcome, metastatic potential and chemoresistance [156,157]. Overexpression of NEDD9 activates Wnt/ß-catenin signaling pathway in CRC [157]. In addition, NEDD9 causes metastatic behavior by promoting E-cadherin removal from the cell membrane and lysosomal degradation [158]. Moreover, butyrate inhibits migration of CRC cells by enhanced expression of miR-200c and the decline in the level of its target BMI1 [159]. BMI1 belongs to polycomb group, and is associated with EMT, invasion and migration of cancer stem cells and chemoresistance; its lower expression is related to longer survival and favorable outcome in CRC patients [160]. Ali et al. revealed that butyrate can also induce expression of miR-139 and miR-542 in CRC cells, which act as suppressor miRNAs through silencing of eukaryotic translation initiation factor 4 gamma 2 (EIF4G2) and baculoviral inhibitor of apoptosis repeat-containing 5 (BIRC5, survivin), respectively [161].

Angiogenesis, an initial stage of new blood vessel formation enhances tumor growth and metastasis mainly through a core regulator of this process, vascular endothelial growth factor (VEGF). VEGF is upregulated in different solid tumor such as primary and metastatic CRC [156]. Angiogenesis in CRC is induced by hypoxia inducible factor 1α (HIF-1α) through the activation of transcription of VEGF [162]. Butyrate has been shown to repress angiogenesis in vitro by diminished presence and activity of pro-angiogenic factor HIF-1α [163]. CRC displays hypoxic areas and activates HIF-1α [164]. Butyrate mediates in HIF-1α activity by decreasing its binding capacity and by downregulating the expression of its downstream gene VEGF [163]. Probably the expression of HIF-1α is significantly inhibited by miR-199a which causes reduced proliferation, migration and invasion of CRC in vitro [165]. On the other hand, downregulation of miR-199a-5p caused by secondary bile acid, deoxycholic acid promotes CRC. Enforced expression of miR-199a-5p inhibits expression of its target CAC1 leading to suppression of tumor growth and restoration of the drug sensitivity of CRC cells [166]. Another in vitro study noticed decreased methylation of miR-126 gene promoter in CRC caused by PUFAs. PUFAs enhance expression of miR-126, lower expression of VEGF and thus showing anti-angiogenic effect [167].

Some of probiotics represent anti-CRC activity, because they remove carcinogens, release antimicrobial and anticarcinogenic agents, improve intestinal permeability, tight junction functions and enzymes activity [168]. Despite these health benefits to the host, important factors such as strains, variability between individuals, location in the intestine and type of administration should be considered [169]. Findings published by Vahed et al. demonstrated that strains of lactic acid bacteria *Leuconostoc mesenteroides*, isolated from traditional dairy products co-cultured with HT-29 cells downregulate levels of two oncomirs miR-21 and miR-200b that promote apoptosis of CRC cells [170]. Programmed cell death is a consequence of upregulation of mitogen-activated protein kinase 1 (MAPK1), Bcl-2-associated protein 4 (Bax) and caspase 3, and downregulation of Akt, NF-κB, Bcl-_XL_ [170]. *L. mesenteroides* belongs to Firmicutes phylum, and *L. mesenteroides* EH-1 strain also produces high concentrations of butyrate in vivo. Butyric acid decreases the rate of *de novo* lipogenesis in a dose dependent manner in vivo. *L. mesenteroides* EH-1 strain activates free fatty acid receptor type 2 (Ffa2), reduces abdominal fats in mice on high-fat diet, and downregulates peroxisome proliferator-activated receptor gamma (PPAR-gamma) which induces differentiation in adipocytes [171]. Obesity, is a crucial environmental risk factor for the pathogenesis of CRC [172]. The metabolism of white adipose tissue (WAT) is under control of gut microbiota, and tryptophan-derived metabolites that are produced by microorganisms are negative regulators of miR-181 family in white adipocytes and contribute to obesity, insulin resistance and inflammation of WAT [173]. Tryptophan is a crucial modulator of immunity and inflammation, and their metabolites, rate-limiting tryptophan metabolizing enzymes and aromatic hydrocarbon receptor are involved in the development of CRC [174].

Reduction of protective bacteria increases the number of other bacterial genera, for example *Bacteroides*, *Prevotella* and *Fusobacterium* that exhibits pro-inflammatory and pro-carcinogenic properties and thus have impact on CRC development [175,176]. Although the main bacteria species involved in CRC pathogenesis are not well understood, it is known that a decrease in bacterial diversity is associated with the relevance to cancer [168]. Yuan et al. identified 76 differentially expressed miRNAs in CRC tissue samples in comparison to adjacent normal colon. Upregulated oncogenic miRNAs identified in cancer tissues are: miR-182, miR-183, miR-503 and miR-17~92 cluster. Moreover, authors showed miRNAs were significantly correlated with the abundance of bacterial genera in CRC microenvironment such as: *Providencia*, *Akkermansia*, *Bacteroides*, *Porphyromonas*, *Roseburia*, *Peptostreptococcus* and *Fusobacterium.* Among these microorganisms, *Akkermansia* spp. is the only taxon correlated with miRNAs involved in CRC pathway [96]. In addition, targets of miRNAs associated with *Fusobacterium* spp. are involved in glycan biosynthesis pathway that probably increases production of glycan by CRC cells, recruits pathogenic bacteria which attached to cells via Fap2 protein and promotes progression of CRC [96]. *Fusobacterium* spp. contribute to the development of CRC not only through virulence factors, metabolites that modulates host immune system such as butyrate, bile acids, retinoic acid, L-tryptophan and its degradation product indole, but also through signaling pathways (Toll-like receptors—TLRs, Nod-like receptors) [77,177]. TLRs play key role in microbe-host interactions, and TLR pathway is dependent or independent on myeloid differentiation factor 88 (MyDD88) adaptor proteins which in turn activates downstream factors, including MAPK, interferon (IFN) and NF-κB [178]. It was shown that *Fusobacterium nucleatum*-mediated infection induces TLR4/MyDD88, activates NF-κB which binds to miR-21 promoter and increases its transcription [51]. Oncogenic roles of miR-21 are related to tumor progression, proliferation, EMT, metastasis, invasion, inhibition of apoptosis and induction of stemness [179]. Moreover, other studies showed *KRAS* mutations were more frequent in *F. nucleatum*-infected CRC which is associated with overexpression of miR-21 as well [180]. Another two miRNAs: miR-34a and miR-135b were upregulated in CRC in comparison to respective normal tissues, and analysis of mRNA:miRNA interactions showed upregulation of miR-34a in CRC due to the TLR2/TLR4-dependent response to *F. nucleatum* [180]. *F. nucleatum*-induced genomic loss of miR-18a* and miR-4802 depends on MyDD8 and TLR4 signaling pathway and these miRNAs target autophagy components, ULK1 and ATG7, respectively, which promote chemoresistance in patients with CRC [181]. Other studies have suggested that upregulation of miR-4474 and miR-4717 in CRC tissues in response to infection with *F. nucleatum* decreases expression of CREB-binding protein (CREBBP) and may promote progression of CRC [182]. CREBBP, a histone acetyltransferase, has a role in transactivation and repression of different genes by acetylation of histone and non-histone proteins [183]. The role of CREBBP in CRC tumorigenesis is not clear, however CREBBP expression was correlated with improved-long term outcomes in patients after primary resection of the tumor (mesorectal excision—TME and partial mesorectal excision—PME), followed by adjuvant chemotherapy (oxaliplatin, folinic acid and 5-fluorouracil) [184].

Exosomes are types of extracellular vesicles that transfer lipids, proteins, nucleic acids, including miRNAs and play a vital role in tumor progression and metastasis [185]. Numerous reports have shown the pivotal role of exosomes in cancer, but their role in bidirectional communication between microbiota and host organism is still analyzed. MiRNAs delivered in exosomes: miR-1246, miR-92b-3p, miR-27a-3p from *F. nucleatum*-infected CRC cells into non-infected cells increases tumor metastasis [186]. Microbes that reside may take up miRNAs which affect the biology of microorganisms. Zhao et al. confirmed that miR-139-5p acts as a tumor suppressor in CRC, and endogenous and exogenous miR-139-5p inhibit proliferation and migration of *F. nucleatum*-infected CRC cells [187]. Another study showed that not only culturing bacteria with host miRNAs affected bacteria growth, but also oral administration of synthetic miRNAs mimics affected specific bacteria in the gut [188].

Food-derived miRNAs (xenomirs) deserve a special attention as a cross-kingdom regulators of gene expression influencing interactions between mammals and microorganisms. Dietary plant-derived miRNAs modulate composition of gut microbiota and regulate intestinal permeability [189]. Teng et al. established plant-derived exosome-like nanoparticles containing miRNAs which may shape expression of genes in the gut microbiota. Exosome-like nanoparticles with mdo-miR-7267-3p target monooxygenase ycnE in *Lactobacillus rhamnosus* which increases indole-3-carboxaldehyde level, a tryptophan metabolite in *L. rhamnosus.* Indole-3-carboxaldehyde, an aryl hydrocarbon receptor ligand, induces expression of pro-inflammatory cytokine IL-22 that inhibits mouse colitis. In addition, metabolic products from plant-derived exosomes inhibit growth of *E. coli*, *Bacteroides fragilis and Listeria* spp. without influence on *L. rhamnosus.* Other plant-derived miRNAs are gma-miR-396e, which promotes growth of *L. rhamnosus* through inhibition of LexA expression, and ath-miR-167a, a key regulator of SpaC expression. Downregulation of SpaC prevents migration of *L. rhamnosus* to the peripheral blood and allows it to remain on the surface of mucosa [190]. It was reported that dietary bovine milk exosomes modulate gut microbiota in mice as well. MiRNAs included in these vesicles stimulate the growth of *Tenericutes*, *Firmicutes* and *Lachnospiraceae* [191]. For sure modulation of microbial physiology by food-derived miRNAs require further analysis, although there is a strong evidence that miRNAs affect intestinal microbiota. Dysbiosis, as a possible consequence of miRNAs-microbiota interactions may be an initial step in CRC development.

The summary of relationships between diet, gut microbiota, bacterial metabolites, miRNAs and their influence on CRC pathogenesis is presented in Table 1.

## 6. Perspectives

A conventional nutritional approach with application of a diet rich in fruits, vegetables and whole grains to balance the gut microbiota may improve health outcomes in individuals with cancer. Transformation of dietary compounds by the gut microbiome may influence epigenetic mechanisms of gene expression as an additional environmental factor. In the human colon microbial metabolites may produce immediate effect, while simultaneously many of them, after absorption into systemic circulation, may alter gene expression in regions distal to the gut. Integration of dietary intake, examination of the gut microbiome and epigenome markers in human population surveys are needed to understand the influence of these environmental factors on human health. Eran Elinav [204] envision the future of cancer care as involving a holistic treatment approach personalized to patient genetic and microbiome characteristics. Undoubtedly, involvement of gut microbiota species in carcinogenesis or in modulation of treatment efficacy is a major premise for new interventions altering microbiota composition and function.

## Figures and Tables

**Table 1:** Interactions between nutrition, intestinal microbiota and miRNAs expression in CRC.

Diet	Link to Microbiome	Link to Epigenetic	Molecular Effects	Reference
High-fiber	Increases level of SCFAs (especially butyrate) produced by *F. prausnitizii* and *Roseburia* spp.	Butyrate inhibits HDAC; Decreases members of miR-106b family (miR-17, miR-20a, miR-20b miR-93, miR-106a, miR-106b)	Butyrate induces CDKN1A (p21) showing anti-proliferative effects of HCT116 cells	[192]
		Butyrate suppresses miR-17-92a cluster, precursor and mature miR-92a; Reduces levels of other members of this cluster: miR-17, -18a, -19a/b	Butyrate reduces c-Myc and augments CDKN1C (p57) levels in HCT116 and HT29 cells	[153]
		Butyrate reduces miR-135a, miR-135b, miR-24, let-7a, miR-106b	Butyrate induces CDKN1A (protein and mRNA levels); Increases cyclin D1 levels (mRNA); Inhibits proliferation of LT97 cells	[193]
		Butyrate induces miR-139 and miR-542	Butyrate inhibits cell-cycle related genes EIF4G2 and BIRC5; Reduces CRC cell proliferation; Induces apoptosis of HCT116 cells	[161]
	Sodium butyrate (NaB)	NaB upregulates miR-200c	NaB inhibits migration of HCT116 and LOVO CRC cells by downregulating of BMI; It reduces cell proliferation, enhances apoptosis and cell cycle arrest	[159]
		NaB increases miR-203	miR-203 reduces NEDD9; Induces CRC apoptosis; Inhibits CRC cell proliferation, colony formation and invasion of HT29 and Caco-2 cells	[194]
		NaB decreases miR-17-92 cluster	Increase in miR-17-92 target genes: PTEN, BCL2L11, CDKN1A; Inhibits proliferation of HT29 and HTC116 cells	[195]
PUFAs	PUFAs correlate with microbiome diversity particularly with bacteria of the *Lachnospiraceae* family	Omega-3 PUFA: docosahexaenoic acid (DHA) increases DNA demethylation of miR-126 promoter and significantly increases expression of miR-126	miR-126 may target VEGF in Caco2 and HCT116 cells; omega-3 PUFAs have anti-angiogenic potential	[167,196]
	High-fat diet increases Firmicutes abundance; Corn oil rich in omega-6 PUFAs increases of *Turicibacteracea* and *Coprococcus* spp.	Corn oil in the presence of carcinogen (AMO) downregulates miR-18a, miR-19b, miR-27b, miR-93, miR-497	Increase of miR-19b in rats treated with AMO and PUFAs downregulates IGF1R levels; Dietary lipids inhibit AMO-induced tumorigenesis: increase apoptosis and suppress cell proliferation in rats	[197,198]
	Consumption of fish oil rich in omega-3 PUFAs modulates intestinal flora through decreasing the growth of enterobacteria and increasing the growth of bifidobacteria; Omega-3 PUFAs alter abundance of *Akkermansia* spp.	Fish oil in the presence of AMO downregulates miR-18a, miR-19b, miR-27b, miR-93	Downregulated miRNAs in rats have predicted targets that are involved in pathways related to CRC: ERK-MAPK, Wnt/ß-catenin, PTEN, apoptosis	[197,199]
PUFAs and high-fiber diet	Pleiotropic effect of fermentable fiber (butyrate) produced by gut microbiota and fatty acids from the diet	Chemoprotective fish oil and pectin-containing diet in the presence of AMO downregulate oncogenic targets of miR-16, miR-21 miR-26b, miR-27b	Diet components reduce expression of these miRNAs in rats; Their molecular targets are involved in apoptosis, mTOR and PI3/AKT signaling	[197]
		Corn oil and cellulose in the presence of AMO downregulate miR-19b, miR-16, miR-27b, miR-203	Increase of TCF4 mRNA level; TCF4 suppresses Wnt/ß-catenin pathway; TCF4 (predicted target for miR-203), PTK2B (predicted target for miR-19b), PDE4B2 (predicted target for miR-26b) are increased in rats	[197]
High-fat, low fiber diet	Increased presence of Gram-negative, proinflammatory genera *Fusobacterium* and *Porphyromonas*; *F. nucleatum* stimulates growth of CRC cells through its unique FadA adhesin; The increased FadA expression in CRC correlates with increased expression of oncogenic and inflammatory genes; Inverse ratio of *Bifidobacterium* spp. to *Escherichia coli*	miR-515-5p and miR-1226-5p enter *F. nucleatum* and *E. coli* and affect their growth and thereby manipulate the diversity of the gut microbiota	*F. nucleatum* via FadA modulates E-cadherin/β-catenin signaling; elevated TNF-α expression in the colon, TNF-α-suppressed differentiation and potentiated cell death induced by butyrate in both adenocarcinoma HT-29 and fetal FHC human colon cells	[77,78,82,200]
High-fat meat	Bile acids enter the colon and are metabolized by microbiota DCA	Decreasing miR199a-5p expression which suppresses tumor cell growth	Increase of CAC1 expression; DCA activates signaling pathways involved in cell proliferation and apoptosis	[201]
Red meat	Heme iron plays role in miRNA processing, alters gene expression and proliferation of colonic epithelium	Induces oncogenic miRNAs: miR-21 and miR-17-92	Decreases CDKN1A (target of miR-17-92); Increases proliferation of colon cells obtained from biopsy samples from healthy individuals	[202]
Red meat with butyrylated resistant starch	Resistant starch fermentation produces butyrate	Restores miR-17-92 but not miR-21	Proliferation of colon cells obtained from biopsy samples from healthy individuals is not completely restored	[202]
Tryptophan-rich diet	Gut bacteria metabolize tryptophan to indicant, indole and indole acid derivatives	Tryptophan-derived metabolites downregulate miR-181 family	Reduced miR-181 level in white adipocyte tissue of obese mice increases the risk of CRC development	[173,203]
Plant diet	Ginger-derived exosome-like particles shape the composition of gut microbiota, particularly are taken up by *Lactobacillaceae*; Metabolic products from plant-derived exosomes inhibit growth of *E. coli*, *Bacteroides fragilis* and *Listeria* spp. without influence on *L. rhamnosus*	Exosome-like nanoparticles contain mdo-miR-7267-3p	mdo-miR-7267-3p targets ycnE monooxygenase yielding indole-3-carboxaldehyde, tryptophan metabolite in *L rhamnosus*; indole as a ligand induces expression of IL-22 and inhibits colitis in mice	[190]
		Exosome-like nanoparticles contain gma-miR-396e	gma-miR-396e promotes growth of *L. rhamnosus* through inhibition of LexA expression	
		Exosome-like nanoparticles contain ath-miR-167	Downregulation of SpaC prevents migration of *L. rhamnosus* to the peripheral blood; Allows bacteria to remain on the surface of mucosa	
	Anticancer effect of urolithins, gut microbiota-derived metabolites of ellagitannins from, i.e., pomegranate	Up-regulation of miR-215 and down-regulation of miR-224	miR-224 is affected by urolithins in SW480 and HT29 cell lines; Upregulation of miR-215 in HT29 cells by urolithins in association with the TP-53 up-regulation of *CDKN1A* gene confirming anti-proliferative and cycle blocking effects of microbial metabolites	[142]
Dairy products	Presence of lactic acid bacteria (*Leuconostoc mesenteroides*, member of *Firmicutes* phylum)	Downregulation of miR-21 and miR-200b	Dairy-isolated probiotic *L. mesenteroides* co-cultured with HT29 CRC cells promotes apoptosis, shows anti-inflammatory and anti-proliferative effects by modulating NF-κB/AKT/PTEN/MAPK pathways; Acts as anti-oncomiRNA in CRC cells	[170]
Bovine milk	Milk-derived exosomes stimulate the growth of *Tenericutes*, *Firmicutes* and *Lachnospiraceae* (Firmicutes phylum)	Exosomes contain miRNAs	Milk exosomes alter gut microbes in murine cecum	[191]

CRC—colorectal cancer; HDAC—histone deacetylase; SCFAs—short-chain fatty acids; PUFAs—polyunsaturated fatty acids; AMO—azoxymethane; DCA—deoxycholic acid.

## Data Availability

No new data were created and analyzed in this manuscript. Data sharing is not applicable.

## Supplementary Materials

The following are available online at https://www.mdpi.com/article/10.3390/nu13072428/s1, Table S1: Examples of oncomirs implicated in colorectal cancer (CRC), Table S2: Examples of suppressor miRNAs involved in colorectal cancer (CRC).
