# Supplementary material for: A Crosstalk between Diet, Microbiome and microRNA in Epigenetic Regulation of Colorectal Cancer
Source: Nutrients. 2021 Jul 15;13(7):2428. doi: 10.3390/nu13072428 (PMC8308570; doi:10.3390/nu13072428)
Supplement: Supplementary file 1 [file nutrients-13-02428-s001.zip › nutrients-1259671-SI.pdf]

Table S1. Examples of oncomirs implicated in colorectal cancer (CRC).

Oncomirs (high expression in CRC)			
MiRNA	Target	Function	Reference
miR-20a	ASK1	Resistance to cisplatin	[1]
miR-21	PNRC2	Stimulates proliferation, invasion, migration, EMT	[2]
	DUSP8	Stimulates proliferation and migration	[3]
	RASA1	Induces proliferation and migration	[4]
miR-22-3p	TLR2	Increases expression of inflammatory mediators	[5]
miR-26b	PTEN, WNT5A	Promotes metastasis	[6]
miR-27a-3p	GSK3β	Promotes metastasis	[7]
miR-28-5p	TLR4	Increases expression of inflammatory mediators	[5]
miR-32	PTEN	Enhances cell proliferation, migration, and invasion; attenuates apoptosis	[8]
	BMP5	Promotes cell proliferation and migration	[9]
	TOB1	Attenuates radiosensitivity; stimulates migration and invasion	[10]
miR-34a-5p	TLR4	Increases expression of inflammatory mediators	[5]
miR-92a	SMAD7, DKK3, KLF4	Enhances proliferation, migration, and invasion	[11]
	p57	Stimulates cell proliferation; inhibits apoptosis	[12]
miR-92b-3p	GSK3β	Promotes metastasis	[7]
miR-106b	PDCD4	Promotes EMT-mediated migration, invasion, and metastasis	[13]
miR-128-3p	FOXO4	Promotes EMT	[14]
miR-135b	TXNIP	Stimulates proliferation, apoptosis, migration, invasion, and angiogenesis	[15]
	IL-6	Increases expression of inflammatory mediators	[5]
miR-142-5p	KLF-6	Stimulates proliferation and migration	[16]
miR-146b	FAM107A	Enhances proliferation, migration, and invasion	[17]
miR-150-5p	TP53	Stimulates proliferation and cell cycle progression; inhibits apoptosis	[18]
miR-181a	SRCIN1	Increases angiogenesis	[19]
miR-182	DAB2IP	Promotes proliferation, invasion and tumor growth	[20]
	FOXO3A	Stimulates cell cycle progression	[21]
miR-183-5p	SOC3	Stimulates proliferation and cell cycle progression; inhibits apoptosis	[22]
	ATG5	Increases radioresistance	[23]
miR-202-5p	PTEN	Enhances cell proliferation and cell cycle progression	[24]
miR-203a-3p	PDE4D	Promotes proliferation and migration	[25]
miR-208b	PDCD4	Promotes tumor growth and reduces oxaliplatin sensitivity	[26]
miR-210-3p	CELF2	Promotes cell cycle progression and proliferation	[27]
miR-222	ATF3	Enhances tumor growth and immune escape	[28]
miR-325-3p	CK18	Promotes EMT	[29]
miR-335-5p	RASA1	Promotes migration, invasion, and EMT	[30]
miR-361-3p	TRAF3	Inhibits apoptosis and facilitates proliferation	[31]
miR-372-3p	LATS2	Promotes proliferation, invasion, and migration	[32]
miR-424-5p	SCN4B	Promotes proliferation and metastasis	[33]
miR-452-5p	PKN2, DUSP6	Inhibits apoptosis; promotes proliferation, and fluorouracil resistance	[34]
miR-454-3p	PTEN	Oxaliplatin resistance	[35]
miR-494	APC	Facilitates proliferation	[36]
miR-503-5p	PDCD4	Induces proliferation, invasion, and migration; inhibits apoptosis	[37]
miR-505	RASSF8	Methotrexate resistance; stimulates migration and invasion; inhibits apoptosis	[38]
miR-532	NKD1	Promotes invasion and metastasis	[39]
miR-543	PLAS3	Enhances migration and invasion	[40]
	PTEN	Oxaliplatin resistance	[41]
miR-574-3p	TGF- β1	Reduces apoptosis; increases proliferation	[42]
miR-592	SPARC	Promotes proliferation, invasion, and migration	[43]
miR-629-5p	CXXC4	Stimulates proliferation and migration; represses apoptosis and fluorouracil sensitivity	[44]

miR-645	EFNA5	Stimulates tumor growth, invasiveness, and metastasis	[45]
miR-663b	TNK1	Enhances stemness and proliferation	[46]
miR-744	BIN1	Oxaliplatin resistance	[47]
miR-885-5p	SOCS5, SOCS6, SOCS7	Stimulates proliferation, migration, and EMT	[48]
miR-942-5p	BATF2	Stimulates colony-forming ability, migration, and invasion; reduces apoptosis	[49]
miR-1246	GSK3 β	Promotes metastasis	[7]
miR-4449	SOCS3	Stimulates proliferation; inhibits apoptosis	[50]
miR-4474	CREBP	Promotes metastasis	[51]
miR-4717	CREBP	Promotes metastasis	[51]

Table S2. Examples of suppressor miRNAs involved in colorectal cancer (CRC).

Suppressor miRs (low expression in CRC)			
MiRNA	Target	Function	Reference
miR-1-3p	YWHAZ	Inhibits proliferation and metastasis	[52]
miR-9-5p	STMN1	Inhibits migration and invasion; stimulates apoptosis	[53]
	ATF1	Inhibits proliferation, migration, and invasion; stimulates apoptosis	[54]
miR-10b	FGF13	Inhibits growth, colony formation, migration and invasion	[55]
	PIK3CA	Activates cancer associated fibroblasts	[56]
miR-18a*	ULK1	Oxaliplatin and fluorouracil sensitivity	[57]
miR-19a	KRAS	Reduces vessels formation and tumor size	[58]
miR-26a-3p	SRSF6	Inhibits proliferation and stimulate apoptosis	[59]
miR-29a-3p	RPS15A	Inhibits proliferation; induces cell cycle arrest and apoptosis	[60]
miR-29b	SIRT1	Oxaliplatin sensitivity	[61]
miR-30c	BCL9	Inhibits proliferation	[62]
miR-34a	LOXL3	Inhibits EMT, migration and invasion	[63]
miR-34b	SOX4	Reduces invasion and migration	[64]
miR-93	HMGB3	Reduces proliferation, invasion, migration, and increases apoptosis	[65]
miR-93-5p	TGF-β	Inhibits proliferation and invasion, stimulate apoptosis	[66]
miR-101	EZH2	Inhibits migration	[67]
miR-106a	FOXQ1	Oxaliplatin sensitivity	[68]
miR-107	TFR1	Suppresses proliferation, migration, and invasion	[69]
	MCM7	Inhibits proliferation, and promotes apoptosis	[70]
miR-124-3p	CD44	Inhibits proliferation	[71]
	PD-L1	Inhibits proliferation, migration, invasion; induces cell cycle arrest and apoptosis	[72]
miR-125	TAZ	Inhibits proliferation and invasion	[73]
miR-125b-2-3p	WEE1	Decreases proliferation and metastasis, increases oxaliplatin sensitivity	[74]
miR-126-5p	PAK2, FZD3	Inhibits proliferation and promotes apoptosis	[75]
miR-128	B7-H3	Inhibits proliferation and migration	[76]
miR-128-5p	SRSF6	Inhibits proliferation and promotes apoptosis	[59]
miR-129	SOX4	Inhibits proliferation, migration, invasion and EMT	[77]
miR-132	ERK1	Inhibits proliferation; induces apoptosis, and reduces adriamycin resistance	[78]
miR-132-3p	USP11	Suppresses cell growth, metastasis, and promotes apoptosis	[79]
	CREB5	Inhibits proliferation, migration, metastasis, and enhances apoptosis	[80]
miR-138-5p	MACC1	Suppresses proliferation, migration, and invasion; promotes apoptosis	[81]
miR-139	EIF4G2	Reduces proliferation and promotes apoptosis	[82]
miR-139-5p	MET	Reduces proliferation and migration	[83]
miR-140-3p	BCL2, BCL9	Suppresses proliferation, migration, invasion, and promotes apoptosis	[84]
miR-141-3p	ZEB1, ZEB2	Inhibits EMT, migration, and invasion	[85]
	TRAF	Inhibits proliferation, migration, and invasion	[86]
miR-142-3p	CTNNB1	Reduces proliferation and colony formation	[87]
miR-143-3p	KLF5	Reduces proliferation, promotes apoptosis	[88]
miR-144	EZH2	Inhibits invasion	[89]
miR-144-3p	BCL6	Inhibits proliferation and induces cell cycle arrest	[90]

miR-145-5p	CDCA3	Suppresses cell viability, cell cycle progression, migration, invasion, and EMT	[91]
miR-146a	RIPK2, TRAF6, PTGES2	Decreases tumorigenic inflammation	[92]
miR-148a	ERBB3	Inhibits proliferation, migration, and invasion	[93]
miR-150	CTNNB1	Decreases proliferation and tumor size	[94]
miR-150-5p	LRG1	Suppresses proliferation, migration, and EMT; promotes apoptosis	[95]
	VEGF	Suppresses proliferation, migration, invasion and angiogenesis	[96]
miR-185-3p	NRP1	Inhibits migration, invasion, and metastasis	[97]
miR-193a-5p	SENP1	Inhibits proliferation, migration, and invasion	[98]
miR-194-5p	SOX5	Suppresses proliferation, migration, and invasion	[99]
miR-195-5p	FZD4	Inhibits proliferation, migration, invasion, and enhances apoptosis	[100]
miR-199a	HIF-1α	Decreases proliferation, migration, and invasion	[101]
miR-199a-5p	CAC1	Suppresses cell growth and induce chemosensitivity	[102]
miR-200c	BMI1	Inhibits invasion and promotes apoptosis	[103]
miR-202	UHRF1	Inhibits proliferation and invasion	[104]
miR-203	NEDD9	Inhibits proliferation, colony formation, invasion, and induces apoptosis	[105]
	RNF6	Suppresses proliferation and cell cycle progression	[106]
miR-211-5p	ERBB4	Inhibits proliferation, colony formation; induces apoptosis and radiosensitivity	[107]
miR-214-3p	CTNNB1	Suppresses growth, migration and invasion; stimulates apoptosis	[108]
miR-216b	SRPK1	Suppresses proliferation, cell cycle progression, migration, and invasion	[109]
miR-296-5p	STAT3	Inhibits proliferation and promotes apoptosis	[110]
	MSI1	Reduces proliferation; induces cell cycle arrest, apoptosis, and radiosensitivity	[111]
miR-320a	SP1	Inhibits cell growth and invasion	[112]
	GMEB1	Inhibits EMT, migration, and invasion	[113]
miR-325	HSPA12B	Oxaliplatin sensitivity	[114]
miR-328-5p	LOXL2	Fluorouracil sensitivity	[115]
miR-331-3p	NRP2	Suppresses migration, invasion, and EMT	[116]
miR-335-5p	LDHB	Inhibits proliferation, migration, and invasion	[117]
miR-338-5p	PAX5	Inhibits proliferation, migration and invasion; induces apoptosis	[118]
miR-340-5p	ANXA3	Inhibits proliferation, migration, and invasion	[119]
miR-363-3p	CXCL5	Inhibits proliferation, migration, and invasion	[120]
	SPHK2	Inhibits proliferation and metastasis; promotes apoptosis	[121]
miR-375	SP1	Inhibits proliferation	[122]
miR-378a-5p	CDK1	Inhibits proliferation	[123]
miR-381-3p	RAB11FIP2	Reduces proliferation, migration, tumor size, and liver metastasis	[124]
miR-382	KLF12, HIPK3	Inhibits proliferation, migration, and invasion; enhances cisplatin sensitivity	[125]
miR-382-5p	EN2	Inhibits proliferation, migration, invasion, and EMT	[126]
miR-429	HMGB3	Decreases proliferation and increases apoptosis	[127]
miR-431	CUL4B	Inhibits EMT, migration, and invasion	[128]
miR-455-5p	PIK3R1	Inhibits proliferation and migration; promotes apoptosis	[129]
miR-485-3p	PDL1	Inhibits proliferation, migration, invasion, and immune escape; promotes apoptosis	[130]
miR-485-5p	SLC38A1	Inhibits colony formation, migration, invasion, glutaminolysis; induces apoptosis	[131]
miR-490-3p	TNKS2	Suppresses migration and invasion; enhances cisplatin and fluorouracil sensitivity	[132]
miR-491	FOXP4	Inhibits proliferation	[133]
miR-495	SP1, SP3	Reduces proliferation, migration, invasion, angiogenesis, and fluorouracil resistance	[134]
	IL-6	Decreases proliferation, migration, invasion, and tumor size	[135]
miR-497-5p	BTF3	Reduces proliferation, migration, and invasion	[136]
miR-519d	CCND1	Fluorouracil sensitivity	[137]
miR-526b-3p	KLF12	Cisplatin sensitivity	[138]
miR-542	BIRC5	Reduces proliferation; induces cell cycle arrest and apoptosis	[82]
miR-590	CDK1	Reduces viability, cell cycle progression, colony formation, and tumor growth	[139]
miR-615-5p	LARP1	Reduces proliferation, migration and invasion; enhances apoptosis	[140]
miR-646	RAB11FIP2	Reduces proliferation, migration, tumor size, and liver metastasis	[124]
miR-654-3p	SRC	Suppresses proliferation and migration; induces cell cycle arrest and apoptosis	[141]

miR-637	WNT1	Inhibits migration, invasion, and induces apoptosis	[142]
miR-761	HDGF	Suppresses tumor growth and induces apoptosis	[143]
	HDAC1	Inhibits proliferation and invasion	[144]
miR-769	HEY1	Inhibits proliferation, cell cycle progression, and invasion	[145]
miR-802	RAN	Inhibits viability, migration, and invasion	[146]
miR-874-3p	GDPD5	Reduces proliferation; induces apoptosis and ferroptosis	[147]
	FOXM1	Inhibits proliferation, invasion, and glycolysis	[148]
miR-944	GATA6	Suppresses EMT, migration, and invasion	[149]
	FZD7	Doxorubicin sensitivity	[150]
	COP1, MDM2	Inhibits cell cycle progression and tumor growth	[151]
miR-1205	TRIM44	Inhibits proliferation, cell cycle progression, metastasis, and promotes apoptosis	[152]
miR-1225	GPR15	Inhibits proliferation, migration, and invasion; induces apoptosis	[153]
miR-1278	KIF5B, CYP24A1	Inhibits proliferation and migration; increases oxaliplatin and calcitriol sensitivity	[154]
miR-1301	STAT3	Suppresses migration and invasion	[155]
miR-1306	VIM	Inhibits cell cycle, proliferation, migration, and invasion	[156]
miR-3666	SATB2	Inhibits proliferation, migration, and invasion	[157]
miR-4319	ABTB1	Suppresses proliferation, cell cycle progression, migration, and invasion	[158]
miR-4324	HOXB2	Suppresses proliferation, migration, and invasion	[159]
miR-4429	FOXM1	Inhibits proliferation, migration, invasion, and EMT	[160]
miR-4802	ATG7	Oxaliplatin and fluorouracil sensitivity	[57]
miR-6887	MEX3A	Inhibits proliferation, migration, and invasion	[161]

References

1. Zhang, L.; He, L.; Zhang, H.; Chen, Y. Knockdown of MiR-20a Enhances Sensitivity of Colorectal Cancer Cells to Cisplatin by Increasing ASK1 Expression. *Cell. Physiol. Biochem.* **2018**, *47*, 1432–1441, doi:10.1159/000490834.
2. Cai, Y.; Li, Y.; Shi, C.; Zhang, Z.; Xu, J.; Sun, B. LncRNA OTUD6B-AS1 inhibits many cellular processes in colorectal cancer by sponging miR-21-5p and regulating PNRC2. *Hum. Exp. Toxicol.* **2021**, doi:10.1177/0960327121997976.
3. Ding, T.; Cui, P.; Zhou, Y.; Chen, C.; Zhao, J.; Wang, H.; Guo, M.; He, Z.; Xu, L. Antisense Oligonucleotides against miR-21 Inhibit the Growth and Metastasis of Colorectal Carcinoma via the DUSP8 Pathway. *Mol. Ther. Nucleic Acids* **2018**, *13*, 244–255, doi:10.1016/j.omtn.2018.09.004.
4. Yang, Y.; Weng, W.; Peng, J.; Hong, L.; Yang, L.; Toiyama, Y.; Gao, R.; Liu, M.; Yin, M.; Pan, C.; et al. Fusobacterium nucleatum Increases Proliferation of Colorectal Cancer Cells and Tumor Development in Mice by Activating Toll-Like Receptor 4 Signaling to Nuclear Factor−κB, and Up-regulating Expression of MicroRNA-21. *Gastroenterology* **2017**, *152*, 851–866, doi:10.1053/j.gastro.2016.11.018.
5. Proença, M.A.; Biselli, J.M.; Succi, M.; Severino, F.E.; Berardinelli, G.N.; Caetano, A.; Reis, R.M.; Hughes, D.J.; Silva, A.E. Relationship between fusobacterium nucleatum, inflammatory mediators and microRNAs in colorectal carcinogenesis. *World J. Gastroenterol.* **2018**, *24*, 5351–5365, doi:10.3748/wjg.v24.i47.5351.
6. Fan, D.; Lin, X.; Zhang, F.; Zhong, W.; Hu, J.; Chen, Y.; Cai, Z.; Zou, Y.; He, X.; Chen, X.; et al. MicroRNA 26b promotes colorectal cancer metastasis by downregulating phosphatase and tensin homolog and wingless-type MMTV integration site family member 5A. *Cancer Sci.* **2018**, *109*, 354–362, doi:10.1111/cas.13451.
7. Guo, S.; Chen, J.; Chen, F.; Zeng, Q.; Liu, W.L.; Zhang, G. Exosomes derived from Fusobacterium nucleatum -infected colorectal cancer cells facilitate tumour metastasis by selectively carrying miR-1246/92b-3p/27a-3p and CXCL16. *Gut* **2020**, doi:10.1136/gutjnl-2020-321187.
8. Wu, W.; Chen, Y.; Ye, S.; Yang, H.; Yang, J.; Quan, J. Transcription factor forkhead box K1 regulates miR-32 expression and enhances cell proliferation in colorectal cancer. *Oncol. Lett.* **2021**, *21*, doi:10.3892/ol.2021.12668.
9. Chen, E.; Li, Q.; Wang, H.; Zhang, P.; Zhao, X.; Yang, F.; Yang, J. MiR-32 promotes tumorigenesis of colorectal cancer by targeting BMP5. *Biomed. Pharmacother.* **2018**, *106*, 1046–1051, doi:10.1016/j.biopha.2018.07.050.
10. Liang, H.; Tang, Y.; Zhang, H.; Zhang, C. Mir-32-5p regulates radiosensitization, migration and invasion of colorectal cancer cells by targeting tob1 gene. *OncoTargets Ther.* **2019**, *12*, 9651–9661, doi:10.2147/OTT.S228995.
11. Chen, E.; Li, Q.; Wang, H.; Yang, F.; Min, L.; Yang, J. MiR-92a promotes tumorigenesis of colorectal cancer, a transcriptomic and functional based study. *Biomed. Pharmacother.* **2018**, *106*, 1370–1377, doi:10.1016/j.biopha.2018.07.098.
12. Hu, S.; Liu, L.; Chang, E.B.; Wang, J.Y.; Raufman, J.P. Butyrate inhibits pro-proliferative miR-92a by diminishing c-Myc-induced miR-17-92a cluster transcription in human colon cancer cells. *Mol. Cancer* **2015**, *14*, doi:10.1186/s12943-015-0450-x.

13. Yang, C.; Dou, R.; Wei, C.; Liu, K.; Shi, D.; Zhang, C.; Liu, Q.; Wang, S.; Xiong, B. Tumor-derived exosomal microRNA-106b-5p activates EMT-cancer cell and M2-subtype TAM interaction to facilitate CRC metastasis. *Mol. Ther.* **2021**, doi:10.1016/j.ymthe.2021.02.006.
14. Bai, J.; Zhang, X.; Shi, D.; Xiang, Z.; Wang, S.; Yang, C.; Liu, Q.; Huang, S.; Fang, Y.; Zhang, W.; et al. Exosomal miR-128-3p Promotes Epithelial-to-Mesenchymal Transition in Colorectal Cancer Cells by Targeting FOXO4 via TGF-β/SMAD and JAK/STAT3 Signaling. *Front. Cell Dev. Biol.* **2021**, *9*, doi:10.3389/fcell.2021.568738.
15. Yin, H.; Yu, S.; Xie, Y.; Dai, X.; Dong, M.; Sheng, C.; Hu, J. Cancer-associated fibroblasts-derived exosomes upregulate microRNA-135b-5p to promote colorectal cancer cell growth and angiogenesis by inhibiting thioredoxin-interacting protein. *Cell. Signal.* **2021**, *84*, 110029, doi:10.1016/j.cellsig.2021.110029.
16. Islam, F.; Gopalan, V.; Vider, J.; Lu, C. tai; Lam, A.K.Y. MiR-142-5p act as an oncogenic microRNA in colorectal cancer: Clinicopathological and functional insights. *Exp. Mol. Pathol.* **2018**, *104*, 98–107, doi:10.1016/j.yexmp.2018.01.006.
17. Wang, D.; Feng, M.; Ma, X.; Tao, K.; Wang, G. Transcription factor SP1-induced microRNA-146b-3p facilitates the progression and metastasis of colorectal cancer via regulating FAM107A. *Life Sci.* **2021**, *277*, 119398, doi:10.1016/j.lfs.2021.119398.
18. Liu, F.; Di Wang, X. miR-150-5p represses TP53 tumor suppressor gene to promote proliferation of colon adenocarcinoma. *Sci. Rep.* **2019**, *9*, doi:10.1038/s41598-019-43231-5.
19. Sun, W.; Wang, X.; Li, J.; You, C.; Lu, P.; Feng, H.; Kong, Y.; Zhang, H.; Liu, Y.; Jiao, R.; et al. MicroRNA-181a promotes angiogenesis in colorectal cancer by targeting SRCIN1 to promote the SRC/VEGF signaling pathway article. *Cell Death Dis.* **2018**, *9*, doi:10.1038/s41419-018-0490-4.
20. Li, X.; Zhang, X.; Zhang, Q.; Lin, R. miR-182 contributes to cell proliferation, invasion and tumor growth in colorectal cancer by targeting DAB2IP. *Int. J. Biochem. Cell Biol.* **2019**, *111*, 27–36, doi:10.1016/j.biocel.2019.04.002.
21. Zhao, H.; Yi, B.; Liang, Z.; Phillips, C.N.; Lin, H.Y.; Riker, A.I.; Xi, Y. Cyclin G2, a novel target of sulindac to inhibit cell cycle progression in colorectal cancer. *Genes Dis.* **2021**, *8*, 320–330, doi:10.1016/j.gendis.2020.11.006.
22. Tu, C.; Wang, Y.; Cheng, X.; Zhu, Y.; Yuan, W.; Dong, J. The combination therapy of fluorouracil and oxaliplatin suppress the progression of colon cancer through mir-183-5p/socs3 axis and downregulating pd-l1. *Cancer Manag. Res.* **2021**, *13*, 1999–2008, doi:10.2147/CMAR.S281925.
23. Zheng, S.; Zhong, Y.F.; Tan, D.M.; Xu, Y.; Chen, H.X.; Wang, D. miR-183-5p enhances the radioresistance of colorectal cancer by directly targeting ATG5. *J. Biosci.* **2019**, *44*.
24. Huang, J.; Zhang, Y.; Xu, Y.; Xie, Q.; Wu, S.; Dong, Y.; Chen, B.; Xia, Y.; Guo, L.; Li, Q.; et al. MiRNA-202-5p promotes Colorectal Carcinogenesis through suppression of PTEN. *J. Cancer* **2021**, *12*, 3154–3163, doi:10.7150/jca.56186.
25. Chen, L.; Gao, H.; Liang, J.; Qiao, J.; Duan, J.; Shi, H.; Zhen, T.; Li, H.; Zhang, F.; Zhu, Z.; et al. miR-203a-3p promotes colorectal cancer proliferation and migration by targeting PDE4D. *Am. J. Cancer Res.* **2018**, *8*, 2387–2401.
26. Ning, T.; Li, J.; He, Y.; Zhang, H.; Wang, X.; Deng, T.; Liu, R.; Li, H.; Bai, M.; Fan, Q.; et al. Exosomal miR-208b Related with Oxaliplatin Resistance Promotes Tregs expansion in Colorectal Cancer. *Mol. Ther.* **2021**, doi:10.1016/j.ymthe.2021.04.028.
27. Ge, L.; Zhou, F.; Nie, J.; Wang, X.; Zhao, Q. Hypoxic colorectal cancer-secreted exosomes deliver miR-210-3p to normoxic tumor cells to elicit a protumoral effect. *Exp. Biol. Med.* **2021**, 153537022110115, doi:10.1177/15353702211011576.
28. Li, S.; Yan, G.; Yue, M.; Wang, L. Extracellular vesicles-derived microRNA-222 promotes immune escape via interacting with ATF3 to regulate AKT1 transcription in colorectal cancer. *BMC Cancer* **2021**, *21*, doi:10.1186/s12885-021-08063-5.
29. Song, C.; Wang, X.; Zhao, X.; Ai, J.; Qi, Y.; Chen, A. MicroRNA-325-3p contributes to colorectal carcinoma by targeting cytokeratin 18. *Oncol. Lett.* **2021**, *21*, doi:10.3892/ol.2021.12509.
30. Sun, X.; Lin, F.; Sun, W.; Zhu, W.; Fang, D.; Luo, L.; Li, S.; Zhang, W.; Jiang, L. Exosome-transmitted miRNA-335-5p promotes colorectal cancer invasion and metastasis by facilitating EMT via targeting RASA1. *Mol. Ther. Nucleic Acids* **2021**, *24*, 164–174, doi:10.1016/j.omtn.2021.02.022.
31. Li, J.; Yang, P.; Chen, F.; Tan, Y.; Huang, C.; Shen, H.; Peng, C.; Feng, Y.; Sun, Y. Hypoxic colorectal cancer-derived extracellular vesicles deliver microRNA-361-3p to facilitate cell proliferation by targeting TRAF3 via the noncanonical NF-κB pathways. *Clin. Transl. Med.* **2021**, *11*, doi:10.1002/ctm2.349.
32. Peng, H.; Pan, X.; Su, Q.; Zhu, L.S.; Ma, G.D. MIR-372-3p promotes tumor progression by targeting LATS2 in colorectal cancer. *Eur. Rev. Med. Pharmacol. Sci.* **2019**, *23*, 8332–8344, doi:10.26355/eurrev_201910_19144.
33. Dai, W.; Zhou, J.; Wang, H.; Zhang, M.; Yang, X.; Song, W. miR-424-5p promotes the proliferation and metastasis of colorectal cancer by directly targeting SCN4B. *Pathol. Res. Pract.* **2020**, *216*, doi:10.1016/j.prp.2019.152731.

34. Lin, X.; Han, L.; Gu, C.; Lai, Y.; Lai, Q.; Li, Q.; He, C.; Meng, Y.; Pan, L.; Liu, S.; et al. MiR-452-5p promotes colorectal cancer progression by regulating an ERK/MAPK positive feedback loop. *Aging (Albany. N. Y.).* **2021**, *13*, 7608–7626, doi:10.18632/aging.202657.
35. Qian, X.-L.; Zhou, F.; Xu, S.; Jiang, J.; Chen, Z.-P.; Wang, S.-K.; Zuo, Y.; Ni, C. MiR-454-3p Promotes Oxaliplatin Resistance by Targeting PTEN in Colorectal Cancer. *Front. Oncol.* **2021**, *11*, 638537, doi:10.3389/fonc.2021.638537.
36. Zhang, Y.; Guo, L.; Li, Y.; Feng, G.H.; Teng, F.; Li, W.; Zhou, Q. MicroRNA-494 promotes cancer progression and targets adenomatous polyposis coli in colorectal cancer. *Mol. Cancer* **2018**, *17*, 1, doi:10.1186/s12943-017-0753-1.
37. Wen, C.; Feng, X.; Yuan, H.; Gong, Y.; Wang, G. Circ_0003266 sponges miR-503-5p to suppress colorectal cancer progression via regulating PDCD4 expression. *BMC Cancer* **2021**, *21*, doi:10.1186/s12885-021-07997-0.
38. Chen, Y.; Bian, L.; Zhang, Y. MiR-505 mediates methotrexate resistance in colorectal cancer by targeting RASSF8. *J. Pharm. Pharmacol.* **2018**, *70*, 937–951, doi:10.1111/jphp.12913.
39. Song, X.; Wu, R.; Tao, Q.; Zhao, L.; Ji, Z. miR-532 promotes colorectal cancer invasion and metastasis by targeting NKD1. *Cell. Mol. Biol.* **2019**, *65*, 52–55, doi:10.14715/cmb/2019.65.6.9.
40. Su, D.W.; Li, X.; Chen, J.; Dou, J.; Fang, G.E.; Luo, C.J. MiR-543 inhibits proliferation and metastasis of human colorectal cancer cells by targeting PLAS3. *Eur. Rev. Med. Pharmacol. Sci.* **2020**, *24*, 8812–8821, doi:10.26355/eurrev_202009_22820.
41. Liang, Y.; Zhu, D.; Zhu, L.; Hou, Y.; Hou, L.; Huang, X.; Li, L.; Wang, Y.; Li, L.; Zou, H.; et al. Dichloroacetate overcomes oxaliplatin chemoresistance in colorectal cancer through the MIR-543/PTEN/AKT/mTOR pathway. *J. Cancer* **2019**, *10*, 6037–6047, doi:10.7150/jca.34650.
42. Li, Y.P.; Du, X.R.; Zhang, R.; Yang, Q. Interleukin-18 promotes the antitumor ability of natural killer cells in colorectal cancer via the miR-574-3p/TGF-β1 axis. *Bioengineered* **2021**, *12*, 763–778, doi:10.1080/21655979.2021.1880717.
43. Pan, Z.; Xie, R.; Song, W.; Gao, C. MicroRNA-592 promotes cell proliferation, migration and invasion in colorectal cancer by directly targeting SPARC. *Mol. Med. Rep.* **2021**, *23*, doi:10.3892/mmr.2021.11900.
44. Lu, J.; Lu, S.; Li, J.; Yu, Q.; Liu, L.; Li, Q. MiR-629-5p promotes colorectal cancer progression through targetting CXXC finger protein 4. *Biosci. Rep.* **2018**, *38*, doi:10.1042/BSR20180613.
45. Li, S.; Hou, X.; Wu, C.; Han, L.; Li, Q.; Wang, J.; Luo, S. MiR-645 promotes invasiveness, metastasis and tumor growth in colorectal cancer by targeting EFNA5. *Biomed. Pharmacother.* **2020**, *125*, doi:10.1016/j.biopha.2020.109889.
46. Hong, S.; Yan, Z.; Wang, H.; Ding, L.; Song, Y.; Bi, M. miR-663b promotes colorectal cancer progression by activating Ras/Raf signaling through downregulation of TNK1. *Hum. Cell* **2020**, *33*, 104–115, doi:10.1007/s13577-019-00294-w.
47. Zhou, Y.; He, A.; Zhang, L.; Yi, G. MiR-744 mediates the oxaliplatin chemoresistance in colorectal cancer through inhibiting BIN1. *Neoplasma* **2020**, *67*, 296–303, doi:10.4149/neo_2019_190508N411.
48. Su, M.; Qin, B.; Liu, F.; Chen, Y.; Zhang, R. Mir-885-5p upregulation promotes colorectal cancer cell proliferation and migration by targeting suppressor of cytokine signaling. *Oncol. Lett.* **2018**, *16*, 65–72, doi:10.3892/ol.2018.8645.
49. Yu, C.; Li, D.; Yan, Q.; Wang, Y.; Yang, X.; Zhang, S.; Zhang, Y.; Zhang, Z. Circ_0005927 inhibits the progression of colorectal cancer by regulating MiR-942-5p/BATF2 axis. *Cancer Manag. Res.* **2021**, *13*, 2295–2306, doi:10.2147/CMAR.S281377.
50. Yan, Z.; Hong, S.; Song, Y.; Bi, M. microR-4449 Promotes Colorectal Cancer Cell Proliferation via Regulation of SOCS3 and Activation of STAT3 Signaling. *Cancer Manag. Res.* **2021**, *13*, 3029–3039, doi:10.2147/cmar.s266153.
51. Feng, Y.Y.; Zeng, D.Z.; Tong, Y.N.; Lu, X.X.; Dun, G.D.; Tang, B.; Zhang, Z.J.; Ye, X.L.; Li, Q.; Xie, J.P.; et al. Alteration of microRNA-4474/4717 expression and CREB-binding protein in human colorectal cancer tissues infected with Fusobacterium nucleatum. *PLoS ONE* **2019**, *14*, e0215088, doi:10.1371/journal.pone.0215088.
52. Du, G.; Yu, X.; Chen, Y.; Cai, W. MiR-1-3p Suppresses Colorectal Cancer Cell Proliferation and Metastasis by Inhibiting YWHAZ-Mediated Epithelial–Mesenchymal Transition. *Front. Oncol.* **2021**, *11*, doi:10.3389/fonc.2021.634596.
53. Bi, C.; Cui, H.; Fan, H.; Li, L. Lncrna linc01116 promotes the development of colorectal cancer by targeting mir-9-5p/stmn1. *OncoTargets Ther.* **2020**, *13*, 10547–10558, doi:10.2147/OTT.S253532.
54. Zhao, X.; Weng, W.; Long, Y.; Pan, W.; Li, Z.; Sun, F. LINC00665/miR-9-5p/ATF1 is a novel axis involved in the progression of colorectal cancer. *Hum. Cell* **2020**, *33*, 1142–1154, doi:10.1007/s13577-020-00393-z.
55. Song, J.J.; Li, W. MiR-10b suppresses the growth and metastasis of colorectal cancer cell by targeting FGF13. *Eur. Rev. Med. Pharmacol. Sci.* **2019**, *23*, 576–587, doi:10.26355/eurrev_201901_16870.
56. Dai, G.; Yao, X.; Zhang, Y.; Gu, J.; Geng, Y.; Xue, F.; Zhang, J. Colorectal cancer cell–derived exosomes containing miR-10b regulate fibroblast cells via the PI3K/Akt pathway. *Bull. Cancer* **2018**, *105*, 336–349, doi:10.1016/j.bulcan.2017.12.009.

57. Yu, T.C.; Guo, F.; Yu, Y.; Sun, T.; Ma, D.; Han, J.; Qian, Y.; Kryczek, I.; Sun, D.; Nagarsheth, N.; et al. Fusobacterium nucleatum Promotes Chemoresistance to Colorectal Cancer by Modulating Autophagy. *Cell* **2017**, *170*, 548–563, doi:10.1016/j.cell.2017.07.008.
58. Chen, M.; Lin, M.; Wang, X. Overexpression of miR-19a inhibits colorectal cancer angiogenesis by suppressing KRAS expression. *Oncol. Rep.* **2018**, *39*, 619–626, doi:10.3892/or.2017.6110.
59. Si, Z.; Yu, L.; Jing, H.; Wu, L.; Wang, X. Oncogenic lncRNA ZNF561-AS1 is essential for colorectal cancer proliferation and survival through regulation of miR-26a-3p/miR-128-5p-SRSF6 axis. *J. Exp. Clin. Cancer Res.* **2021**, *40*, doi:10.1186/s13046-021-01882-1.
60. Zheng, Z.; Cui, H.; Wang, Y.; Yao, W. Downregulation of RPS15A by miR-29a-3p attenuates cell proliferation in colorectal carcinoma. *Biosci. Biotechnol. Biochem.* **2019**, *83*, 2057–2064, doi:10.1080/09168451.2019.1637712.
61. Liu, H.; Cheng, X.H. MiR-29b reverses oxaliplatin-resistance in colorectal cancer by targeting SIRT1. *Oncotarget* **2018**, *9*, 12304–12315, doi:10.18632/oncotarget.24380.
62. Zhao, D.W.; Li, M.M.; Han, J.P.; Wang, Y.; Jiang, L.X.; Chang, H.L. MiR-30c exerts tumor suppressive functions in colorectal carcinoma by directly targeting BCL9. *Eur. Rev. Med. Pharmacol. Sci.* **2019**, *23*, 3335–3343, doi:10.26355/eurrev_201904_17696.
63. Ren, J.; Wang, X.; Wei, G.; Meng, Y. Exposure to desflurane anesthesia confers colorectal cancer cells metastatic capacity through deregulation of miR-34a/LOXL3. *Eur. J. Cancer Prev.* **2021**, *30*, 143–153, doi:10.1097/CEJ.0000000000000608.
64. Deng, H.; Wang, M.; Xu, Q.; Yao, H. ZFAS1 Promotes Colorectal Cancer Metastasis Through Modulating miR-34b/SOX4 Targeting. *Cell Biochem. Biophys.* **2021**, *79*, doi:10.1007/s12013-021-00976-z.
65. Gu, M.; Jiang, Z.; Li, H.; Peng, J.; Chen, X.; Tang, M. MiR-93/HMGB3 regulatory axis exerts tumor suppressive effects in colorectal carcinoma cells. *Exp. Mol. Pathol.* **2021**, *120*, 104635, doi:10.1016/j.yexmp.2021.104635.
66. Li, Q.; Yue, W.; Li, M.; Jiang, Z.; Hou, Z.; Liu, W.; Ma, N.; Gan, W.; Li, Y.; Zhou, T.; et al. Downregulating Long Non-coding RNAs CTBP1-AS2 Inhibits Colorectal Cancer Development by Modulating the miR-93-5p/TGF-β/SMAD2/3 Pathway. *Front. Oncol.* **2021**, *11*, doi:10.3389/fonc.2021.626620.
67. Huang, Z.; Wu, X.; Li, J. miR-101 suppresses colon cancer cell migration through regulation of EZH2. *Rev. Española Enfermedades Dig.* **2020**, *113*, doi:10.17235/reed.2020.6800/2019.
68. Liu, Z.; Qin, Y.; Dong, S.; Chen, X.; Huo, Z.; Zhen, Z. Overexpression of miR-106a enhances oxaliplatin sensitivity of colorectal cancer through regulation of FOXQ1. *Oncol. Lett.* **2020**, *19*, 663–670, doi:10.3892/ol.2019.11151.
69. Fu, Y.; Lin, L.; Xia, L. MiR-107 function as a tumor suppressor gene in colorectal cancer by targeting transferrin receptor 1. *Cell. Mol. Biol. Lett.* **2019**, *24*, doi:10.1186/s11658-019-0155-z.
70. Zhao, M.; Wang, Y.; Jiang, C.; Wang, Q.; Mi, J.; Zhang, Y.; Zuo, L.; Geng, Z.; Song, X.; Ge, S.; et al. miR-107 regulates the effect of MCM7 on the proliferation and apoptosis of colorectal cancer via the PAK2 pathway. *Biochem. Pharmacol.* **2021**, 114610, doi:10.1016/j.bcp.2021.114610.
71. Zhang, H. rong; Wu, S. yong; Fu, Z. xue LncRNA-cCSC1 promotes cell proliferation of colorectal cancer through sponging miR-124-3p and upregulating CD44. *Biochem. Biophys. Res. Commun.* **2021**, *557*, 228–235, doi:10.1016/j.bbrc.2021.04.018.
72. Roshani Asl, E.; Rasmi, Y.; Baradaran, B. MicroRNA-124-3p suppresses PD-L1 expression and inhibits tumorigenesis of colorectal cancer cells via modulating STAT3 signaling. *J. Cell. Physiol.* **2021**, doi:10.1002/jcp.30378.
73. Yang, M.; Tang, X.; Wang, Z.; Wu, X.; Tang, D.; Wang, D. MiR-125 inhibits colorectal cancer proliferation and invasion by targeting TAZ. *Biosci. Rep.* **2019**, *39*, doi:10.1042/BSR20190193.
74. Zeng, Z. lei; Lu, J. huan; Wang, Y.; Sheng, H.; Wang, Y. nan; Chen, Z. hong; Wu, Q. nian; Zheng, J.B.; Chen, Y. xing; Yang, D. dong; et al. The lncRNA XIST/miR-125b-2-3p axis modulates cell proliferation and chemotherapeutic sensitivity via targeting Wee1 in colorectal cancer. *Cancer Med.* **2021**, *10*, 2423–2441, doi:10.1002/cam4.3777.
75. Wu, C.-L.; Shan, T.-D.; Han, Y.; Kong, Y.; Li, Y.-B.; Peng, X.-G.; Shang, L.; Wang, P.-G.; Li, L.-P. Long intergenic noncoding RNA 00665 promotes proliferation and inhibits apoptosis in colorectal cancer by regulating miR-126-5p. *Aging (Albany. N. Y).* **2021**, *13*, doi:10.18632/aging.202874.
76. Hu, X.; Xu, M.; Hu, Y.; Li, N.; Zhou, L. B7-H3, Negatively Regulated by miR-128, Promotes Colorectal Cancer Cell Proliferation and Migration. *Cell Biochem. Biophys.* **2021**, *79*, doi:10.1007/s12013-021-00975-0.
77. Chen, Z.; Zhong, T.; Zhong, J.; Tang, Y.; Ling, B.; Wang, L. MicroRNA-129 inhibits colorectal cancer cell proliferation, invasion and epithelial-to-mesenchymal transition by targeting SOX4. *Oncol. Rep.* **2021**, *45*, doi:10.3892/OR.2021.8012.
78. Liu, Y.; Zhang, M. MiR-132 Regulates Adriamycin Resistance in Colorectal Cancer Cells Through Targeting Extracellular Signal-Regulated Kinase 1. *Cancer Biother. Radiopharm.* **2019**, *34*, 398–404, doi:10.1089/cbr.2018.2749.
79. Zhang, W.; Wang, Z.; Cai, G.; Huang, P. Circ_DOCK1 regulates USP11 through miR-132-3p to control colorectal cancer progression. *World J. Surg. Oncol.* **2021**, *19*, doi:10.1186/s12957-021-02173-x.

80. Zhang, M.; Li, Y.; Wang, H.; Yu, W.; Lin, S.; Guo, J. LncRNA SNHG5 affects cell proliferation, metastasis and migration of colorectal cancer through regulating miR-132-3p/CREB5. *Cancer Biol. Ther.* **2019**, *20*, 524–536, doi:10.1080/15384047.2018.1537579.
81. Wei, J.; Lin, Y.; Wang, Z.; Liu, Y.; Guo, W. Circ_0006174 accelerates colorectal cancer progression through regulating mir-138-5p/ macc1 axis. *Cancer Manag. Res.* **2021**, *13*, 1673–1686, doi:10.2147/CMAR.S295833.
82. Ali, S.R.; Orang, A.; Marri, S.; McKinnon, R.A.; Meech, R.; Michael, M.Z. Integrative transcriptomic network analysis of butyrate treated colorectal cancer cells. *Cancers (Basel)* **2021**, *13*, 1–26, doi:10.3390/cancers13040636.
83. Zhao, Y.; Tao, Q.; Li, S.; Zheng, P.; Liu, J.; Liang, X. Both endogenous and exogenous miR-139–5p inhibit Fusobacterium nucleatum-related colorectal cancer development. *Eur. J. Pharmacol.* **2020**, *888*, doi:10.1016/j.ejphar.2020.173459.
84. Liu, D.; Chen, C.; Cui, M.; Zhang, H. miR-140-3p inhibits colorectal cancer progression and its liver metastasis by targeting BCL9 and BCL2. *Cancer Med.* **2021**, doi:10.1002/cam4.3840.
85. Sun, Z.; Shao, B.; Liu, Z.; Dang, Q.; Guo, Y.; Chen, C.; Guo, Y.; Chen, Z.; Liu, J.; Hu, S.; et al. LINC01296/miR-141-3p/ZEB1-ZEB2 axis promotes tumor metastasis via enhancing epithelial-mesenchymal transition process. *J. Cancer* **2021**, *12*, 2723–2734, doi:10.7150/JCA.55626.
86. Liang, Z.; Li, X.; Liu, S.; Li, C.; Wang, X.; Xing, J. MiR-141–3p inhibits cell proliferation, migration and invasion by targeting TRAF5 in colorectal cancer. *Biochem. Biophys. Res. Commun.* **2019**, *514*, 699–705, doi:10.1016/j.bbrc.2019.05.002.
87. Liu, P.; Cao, F.; Sui, J.; Hong, Y.; Liu, Q.; Gao, X.H.; Gong, H.; Hao, L.; Lou, Z.; Zhang, W. MicroRNA-142-3p Inhibits Tumorigenesis of Colorectal Cancer via Suppressing the Activation of Wnt Signaling by Directly Targeting to β-Catenin. *Front. Oncol.* **2020**, *10*, doi:10.3389/fonc.2020.552944.
88. Shan, T.D.; Tian, Z. Bin; Li, Q.; Jiang, Y.P.; Liu, F.G.; Sun, X.G.; Han, Y.; Sun, L.J.; Chen, L. Long intergenic noncoding RNA 00908 promotes proliferation and inhibits apoptosis of colorectal cancer cells by regulating KLF5 expression. *J. Cell. Physiol.* **2021**, *236*, 889–899, doi:10.1002/jcp.29899.
89. Ma, X.; Lv, L.; Xing, C. Circ_ 0115744 acts as miR-144 sponge to promote and predict the metastasis of colorectal cancer. *Aging (Albany. N. Y.).* **2021**, *13*, 5892–5905, doi:10.18632/aging.202513.
90. Sun, N.; Zhang, L.; Zhang, C.; Yuan, Y. MiR-144-3p inhibits cell proliferation of colorectal cancer cells by targeting BCL6 via inhibition of Wnt/β-catenin signaling. *Cell. Mol. Biol. Lett.* **2020**, *25*, doi:10.1186/s11658-020-00210-3.
91. Chen, Q.; Zhou, L.; Ye, X.; Tao, M.; Wu, J. miR-145-5p suppresses proliferation, metastasis and EMT of colorectal cancer by targeting CDCA3. *Pathol. Res. Pract.* **2020**, *216*, doi:10.1016/j.prp.2020.152872.
92. Garo, L.P.; Ajay, A.K.; Fujiwara, M.; Gabriely, G.; Raheja, R.; Kuhn, C.; Kenyon, B.; Skillin, N.; Kadowaki-Saga, R.; Saxena, S.; et al. MicroRNA-146a limits tumorigenic inflammation in colorectal cancer. *Nat. Commun.* **2021**, *12*, 1–16, doi:10.1038/s41467-021-22641-y.
93. Zhao, W.; Zheng, J.; Wei, G.; Yang, K.; Wang, G.; Sun, X. MiR-148a inhibits cell proliferation and migration through targeting ErbB3 in colorectal cancer. *Oncol. Lett.* **2019**, *18*, 2530–2536, doi:10.3892/ol.2019.10581.
94. He, Z.; Dang, J.; Song, A.; Cui, X.; Ma, Z.; Zhang, Y. The involvement of miR-150/β-catenin axis in colorectal cancer progression. *Biomed. Pharmacother.* **2020**, *121*, doi:10.1016/j.biopha.2019.109495.
95. Lou, T.; Ke, K.; Zhang, L.; Miao, C.; Liu, Y. LncRNA PART1 facilitates the malignant progression of colorectal cancer via miR-150-5p/LRG1 axis. *J. Cell. Biochem.* **2020**, *121*, 4271–4281, doi:10.1002/jcb.29635.
96. Chen, X.; Xu, X.; Pan, B.; Zeng, K.; Xu, M.; Liu, X.; He, B.; Pan, Y.; Sun, H.; Wang, S. miR-150-5p suppresses tumor progression by targeting VEGFA in colorectal cancer. *Aging (Albany. N. Y.).* **2018**, *10*, 3421–3437, doi:10.18632/aging.101656.
97. Liu, X.; Meng, X.; Peng, X.; Yao, Q.; Zhu, F.; Ding, Z.; Sun, H.; Liu, X.; Li, D.; Lu, Y.; et al. Impaired AGO2/miR-185-3p/NRP1 axis promotes colorectal cancer metastasis. *Cell Death Dis.* **2021**, *12*, doi:10.1038/s41419-021-03672-1.
98. Zhou, M.; Bian, Z.; Liu, B.; Zhang, Y.; Cao, Y.; Cui, K.; Sun, S.; Li, J.; Zhang, J.; Wang, X.; et al. Long noncoding RNA MCM3AP-AS1 enhances cell proliferation and metastasis in colorectal cancer by regulating miR-193a-5p/SENP1. *Cancer Med.* **2021**, *10*, 2470–2481, doi:10.1002/cam4.3830.
99. Feng, Y.; Xu, Y.; Gao, Y.; Chen, Y.; Wang, X.; Chen, Z. A novel lncRNA SOX2OT promotes the malignancy of human colorectal cancer by interacting with miR-194-5p/SOX5 axis. *Cell Death Dis.* **2021**, *12*, 499, doi:10.1038/s41419-021-03756-y.
100. Yang Leilei, Bi Tienan, Zhou Shenkang, Lan Yuefu, Z.R. CircRASSF2 facilitates the proliferation and metastasis of colorectal cancer by mediating the activity of Wnt/β-catenin signaling pathway by regulating the miR-195-5p/FZD4 axis. *Anticancer Drugs* **2021**, doi:10.1097/CAD.0000000000001084.
101. Ye, H.; Pang, L.; Wu, Q.; Zhu, Y.; Guo, C.; Deng, Y.; Zheng, X. A critical role of mir-199a in the cell biological behaviors of colorectal cancer. *Diagn. Pathol.* **2015**, *10*, doi:10.1186/s13000-015-0260-x.
102. Dong, J.; Tai, J.W.; Lu, L.F. miRNA–Microbiota Interaction in Gut Homeostasis and Colorectal Cancer. *Trends Cancer* **2019**, *5*, 666–669.

103. Karimi Mazraehshah, M.; Tavangar, S.M.; Saidijam, M.; Amini, R.; Bahreini, F.; Karimi Dermani, F.; Najafi, R. Anticancer effects of miR-200c in colorectal cancer through BMI1. *J. Cell. Biochem.* **2018**, *119*, 10005–10012, doi:10.1002/jcb.27330.
104. Lin, Y.; Chen, Z.; Lin, S.; Zheng, Y.; Liu, Y.; Gao, J.; Chen, S. MIR-202 inhibits the proliferation and invasion of colorectal cancer by targeting UHRF1. *Acta Biochim. Biophys. Sin. (Shanghai)* **2019**, *51*, 598–606, doi:10.1093/abbs/gmz042.
105. Wu, X.; Wu, Y.; He, L.; Wu, L.; Wang, X.; Liu, Z. Effects of the intestinal microbial metabolite butyrate on the development of colorectal cancer. *J. Cancer* **2018**, *9*, 2510–2517.
106. Miao, J.; Hou, N.; Yang, W.; Jiang, Q.; Xue, W.; Wang, X.; Zhang, H.; Xiong, X.; Wang, L.; Zhao, L.; et al. miR-203a suppresses cell proliferation by targeting RING-finger protein 6 in colorectal cancer. *Anticancer. Drugs* **2020**, *31*, 583–591, doi:10.1097/CAD.0000000000000874.
107. Li, C.; Liu, H.; Wei, R.; Liu, Z.; Chen, H.; Guan, X.; Zhao, Z.; Wang, X.; Jiang, Z. LncRNA EGOT/miR-211-5p Affected Radiosensitivity of Rectal Cancer by Competitively Regulating ErbB4. *OncoTargets Ther.* **2021**, *14*, 2867–2878, doi:10.2147/OTT.S256989.
108. Han, T.; Gao, M.; Wang, X.; Li, W.; Zhuo, J.; Qu, Z.; Chen, Y. LINC00665 activates Wnt/β-catenin signaling pathway to facilitate tumor progression of colorectal cancer via upregulating CTNNB1. *Exp. Mol. Pathol.* **2021**, *120*, 104639, doi:10.1016/j.yexmp.2021.104639.
109. Yao, Y.F.; Li, Q.R.; Wang, H. MiR-216b suppresses colorectal cancer proliferation, migration, and invasion by targeting SR PK1. *OncoTargets Ther.* **2018**, *11*, 1671–1681, doi:10.2147/OTT.S161835.
110. Han, W.; Kong, D.; Lu, Q.; Zhang, W.; Fan, Z. Aloperine inhibits proliferation and promotes apoptosis in Colorectal Cancer cells by regulating the circNSUN2/miR-296-5p/STAT3 pathway. *Drug Des. Dev. Ther.* **2021**, *15*, 857–870, doi:10.2147/DDDT.S288473.
111. Li, L.; Jiang, Z.; Zou, X.; Hao, T. Exosomal circ_ift80 enhances tumorigenesis and suppresses radiosensitivity in colorectal cancer by regulating mir-296-5p/msi1 axis. *Cancer Manag. Res.* **2021**, *13*, 1929–1941, doi:10.2147/CMAR.S297123.
112. Zhang, W.; Yang, H.; Wang, Z.; Wu, Y.; Wang, J.; Duan, G.; Guo, Q.; Zhang, Y. miR-320a/SP1 negative reciprocal interaction contributes to cell growth and invasion in colorectal cancer. *Cancer Cell Int.* **2021**, *21*, doi:10.1186/s12935-021-01874-3.
113. Cui, Z.; Sun, Q.; Yan, W.; Han, Q.; Wang, G.; Hu, Y. The role of miR-320a and its target gene GMEB1 in epithelial-mesenchymal transition and invasion of colorectal cancer. *J. Gene Med.* **2021**, doi:10.1002/jgm.3327.
114. Zhang, L.; Chen, H.; Song, Y.; Gu, Q.; Zhang, L.; Xie, Q.; Xu, J.; Zhang, M. MiR-325 Promotes Oxaliplatin-Induced Cytotoxicity Against Colorectal Cancer Through the HSPA12B/PI3K/AKT/Bcl-2 Pathway. *Dig. Dis. Sci.* **2020**, doi:10.1007/s10620-020-06579-7.
115. Zheng, G.-L.; Liu, Y.-L.; Yan, Z.-X.; Xie, X.-Y.; Xiang, Z.; Yin, L.; Wang, Q.-Q.; Chong, D.-C.; Xue, G.-L.; Xu, L.-L.; et al. Elevated LOXL2 expression by LINC01347/miR-328-5p axis contributes to 5-FU chemotherapy resistance of colorectal cancer. *Am. J. Cancer Res.* **2021**, *11*, 1572–1585.
116. Zhang, H.; Wang, R.; Wang, M. MiR-331-3p suppresses cell invasion and migration in colorectal carcinoma by directly targeting NRP2. *Oncol. Lett.* **2019**, *18*, 6501–6508, doi:10.3892/ol.2019.11029.
117. Zhang, D.; Yang, N. MiR-335-5p inhibits cell proliferation, migration and invasion in colorectal cancer through downregulating LDHB. *J. B.U.ON.* **2019**, *24*, 1128–1136.
118. Li, W.; He, Y.; Cheng, Z. Long noncoding RNA XIST knockdown suppresses the growth of colorectal cancer cells via regulating microRNA-338-3p/PAX5 axis. *Eur. J. Cancer Prev.* **2021**, *30*, 132–142, doi:10.1097/CEJ.0000000000000596.
119. Yang, L.; Men, W.L.; Yan, K.M.; Tie, J.; Nie, Y.Z.; Xiao, H.J. MiR-340-5p is a potential prognostic indicator of colorectal cancer and modulates ANXA3. *Eur. Rev. Med. Pharmacol. Sci.* **2018**, *22*, 4837–4845, doi:10.26355/eurrev_201808_15619.
120. Zhang, Y.; Zheng, S.; Liao, N.; Huang, H.; Chen, W.; Wu, Z.; Wu, D. CircCTNNA1 acts as a ceRNA for miR-363-3p to facilitate the progression of colorectal cancer by promoting CXCL5 expression. *J. Biol. Res.* **2021**, *28*, doi:10.1186/s40709-021-00135-8.
121. Dong, J.; Geng, J.; Tan, W. MiR-363-3p suppresses tumor growth and metastasis of colorectal cancer via targeting SphK2. *Biomed. Pharmacother.* **2018**, *105*, 922–931, doi:10.1016/j.biopha.2018.06.052.
122. Liu, X.H.; Wang, J.; Dong, Y.H. The inhibitory effect of miR-375 targeting spi in colorectal cancer cell proliferation. *Eur. Rev. Med. Pharmacol. Sci.* **2018**, *22*, 405–411, doi:10.26355/eurrev_201801_14188.
123. Li, K.; Zhang, J.; Zhang, M.; Wu, Y.; Lu, X.; Zhu, Y. miR-378a-5p inhibits the proliferation of colorectal cancer cells by downregulating CDK1. *World J. Surg. Oncol.* **2021**, *19*, doi:10.1186/s12957-021-02166-w.
124. Zhang, W.; Li, X.; Zhang, W.; Lu, Y.; Lin, W.; Yang, L.; Zhang, Z.; Li, X. The LncRNA CASC11 Promotes Colorectal Cancer Cell Proliferation and Migration by Adsorbing miR-646 and miR-381-3p to Upregulate Their Target RAB11FIP2. *Front. Oncol.* **2021**, *11*, doi:10.3389/fonc.2021.657650.

125. Yao, H.; Xia, D.; Li, Z. lin; Ren, L.; Wang, M. ming; Chen, W. sheng; Hu, Z. chuan; Yi, G. ping; Xu, L. MIR-382 functions as tumor suppressor and chemosensitizer in colorectal cancer. *Biosci. Rep.* **2019**, *39*, doi:10.1042/BSR20180441.
126. Xie, L.; Pan, Z. Circular RNA circ_0000467 regulates colorectal cancer development via miR-382-5p/EN2 axis. *Bioengineered* **2021**, *12*, 886–897, doi:10.1080/21655979.2021.1889130.
127. Tian, X.; Chang, J.; Zhang, N.; Wu, S.; Liu, H.; Yu, J. MicroRNA-429 acts as a tumor suppressor in colorectal cancer by targeting high mobility group box 3. *Oncol. Lett.* **2021**, *21*, doi:10.3892/ol.2021.12511.
128. Su, W.B.; Liu, Z.Y. MiR-431 inhibits colorectal cancer cell invasion via repressing CUL4B. *Eur. Rev. Med. Pharmacol. Sci.* **2018**, *22*, 3047–3052, doi:10.26355/eurrev_201805_15062.
129. Wang, J.; Lu, Y.; Zeng, Y.; Zhang, L.; Ke, K.; Guo, Y.U. Expression profile and biological function of miR-455-5p in colorectal carcinoma. *Oncol. Lett.* **2019**, *17*, 2131–2140, doi:10.3892/ol.2018.9862.
130. Jiang, Z.; Hou, Z.; Liu, W.; Yu, Z.; Liang, Z.; Chen, S. Circ-KRT6C promotes malignant progression and immune evasion of colorectal cancer through miR-485-3p/PDL1 axis. *J. Pharmacol. Exp. Ther.* **2021**, JPET-AR-2021-000518, doi:10.1124/jpet.121.000518.
131. Yu, J.; Chen, X.; Li, J.; Wang, F. CircRUNX1 functions as an oncogene in colorectal cancer by regulating circRUNX1/miR-485-5p/SLC38A1 axis. *Eur. J. Clin. Investig.* **2021**, doi:10.1111/eci.13540.
132. Li, J.; Mo, R.; Zheng, L. MicroRNA-490-3p inhibits migration and chemoresistance of colorectal cancer cells via targeting TNKS2. *World J. Surg. Oncol.* **2021**, *19*, doi:10.1186/s12957-021-02226-1.
133. WU, H.; TAO, Y.; ZHANG, W.; WANG, G.; ZHANG, Q. circ-0000212 promotes cell proliferation of colorectal cancer by sponging miR-491 and modulating FOXP4 expression. *Mol. Med. Rep.* **2021**, *23*, doi:10.3892/MMR.2021.11939.
134. Liu, S.J.; Li, Z.Q.; Wang, X.Y.; Liu, F.; Xiao, Z.M.; Zhang, D.C. lncRNA UCA1 induced by SP1 and SP3 forms a positive feedback loop to facilitate malignant phenotypes of colorectal cancer via targeting miR-495. *Life Sci.* **2021**, *277*, 119569, doi:10.1016/j.lfs.2021.119569.
135. Liu, X.; Qin, Y.; Tang, X.; Wang, Y.; Bian, C.; Zhong, J. Circular RNA circ_0000372 contributes to the proliferation, migration and invasion of colorectal cancer by elevating IL6 expression via sponging miR-495. *Anticancer Drugs* **2021**, *32*, 296–305, doi:10.1097/CAD.0000000000001002.
136. Wang, H.; Xing, J.; Wang, W.; Lv, G.; He, H.; Lu, Y.; Sun, M.; Chen, H.; Li, X. Molecular Characterization of the Oncogene BTF3 and Its Targets in Colorectal Cancer. *Front. Cell Dev. Biol.* **2021**, *8*, doi:10.3389/fcell.2020.601502.
137. Huang, R.; Lin, J.Y.; Chi, Y.J. MiR-519d reduces the 5-fluorouracil resistance in colorectal cancer cells by down-regulating the expression of CCND1. *Eur. Rev. Med. Pharmacol. Sci.* **2018**, *22*, 2869–2875, doi:10.26355/eurrev_201805_14989.
138. Zhang, W.; Wang, Z.; Cai, G.; Huang, P. Downregulation of circ_0071589 suppresses cisplatin resistance in colorectal cancer by regulating the MiR-526b-3p/KLF12 axis. *Cancer Manag. Res.* **2021**, *13*, 2717–2731, doi:10.2147/CMAR.S294880.
139. Zhou, Z.; Tan, F.; Pei, Q.; Li, C.; Zhou, Y.; Li, Y.; Pei, H. LncRNA SNHG4 modulates colorectal cancer cell cycle and cell proliferation through regulating miR-590-3p/CDK1 axis. *Aging (Albany. N. Y.).* **2021**, *13*, doi:10.18632/aging.202737.
140. Wu, M.; Kong, C.; Cai, M.; Huang, W.; Chen, Y.; Wang, B.; Liu, X. Hsa_circRNA_002144 promotes growth and metastasis of colorectal cancer through regulating miR-615-5p/LARP1/mTOR pathway. *Carcinogenesis* **2020**, *42*, doi:10.1093/carcin/bgaa140.
141. Zhang, H.; Shen, Z.; Zhou, Y.; Zhang, Z.; Wang, Q.; Zhang, M.; Jiang, K.; Wang, S.; Ye, Y.; Wang, B. Downregulation of miR-654-3p in Colorectal Cancer Indicates Poor Prognosis and Promotes Cell Proliferation and Invasion by Targeting SRC. *Front. Genet.* **2020**, *11*, doi:10.3389/fgene.2020.577948.
142. Hu, N.; Duan, J.-A.; Yu, Y.; Li, D.; Chen, J.; Yan, H. Sevoflurane inhibits the migration, invasion and induces apoptosis by regulating the expression of WNT1 via miR-637 in colorectal cancer. *Anticancer Drugs* **2021**, *32*, 537–547, doi:10.1097/cad.0000000000001061.
143. Zhang, W.; Liu, Q.; Luo, L.; Song, J.; Han, K.; Liu, R.; Gong, Y.; Guo, X. Use Chou's 5-steps rule to study how Baicalin suppresses the malignant phenotypes and induces the apoptosis of colorectal cancer cells. *Arch. Biochem. Biophys.* **2021**, 108919, doi:10.1016/j.abb.2021.108919.
144. Xiong, W.; Yang, S.; Zhang, W.; Chen, Y.; Wang, F. MiR-761 inhibits colorectal cancer cell proliferation and invasion through targeting HDAC1. *Pharmazie* **2019**, *74*, 111–114, doi:10.1691/ph.2019.8756.
145. Han, C.; Song, Y.; Lian, C. MiR-769 inhibits colorectal cancer cell proliferation and invasion by targeting HEY1. *Med. Sci. Monit.* **2018**, *24*, 9232–9239, doi:10.12659/MSM.911663.
146. Feng, H.; Liu, L.; Xu, L.; Wang, H.; Hua, Q.; He, P. MiR-802 suppresses colorectal cancer cell viability, migration and invasion by targeting RAN. *Cancer Manag. Res.* **2020**, *12*, 2291–2300, doi:10.2147/CMAR.S231709.
147. Wang, Y.; Chen, H.; Wei, X. Circ_0007142 downregulates miR-874-3p-mediated GDPD5 on colorectal cancer cells. *Eur. J. Clin. Investig.* **2021**, doi:10.1111/eci.13541.

148. Zhang, Z.; Yang, W.; Li, N.; Chen, X.; Ma, F.; Yang, J.; Zhang, Y.; Chai, X.F.; Zhang, B.; Hou, X.; et al. LncRNA MCF2L-AS1 aggravates proliferation, invasion and glycolysis of colorectal cancer cells via the crosstalk with miR-874-3p/FOXM1 signaling axis. *Carcinogenesis* **2021**, *42*, 263–271, doi:10.1093/carcin/bgaa093.
149. Tang, J.T.; Gao, W.; Liu, G.; Sheng, W.W.; Zhou, J.P.; Dong, Q.; Dong, M. miR-944 suppresses EGF-induced EMT in colorectal cancer cells by directly targeting GATA6. *OncoTargets Ther.* **2021**, *14*, 2311–2325, doi:10.2147/OTT.S290567.
150. Xi, L.; Liu, Q.; Zhang, W.; Luo, L.; Song, J.; Liu, R.; Wei, S.; Wang, Y. Circular RNA circCSPP1 knockdown attenuates doxorubicin resistance and suppresses tumor progression of colorectal cancer via miR-944/FZD7 axis. *Cancer Cell Int.* **2021**, *21*, doi:10.1186/s12935-021-01855-6.
151. Kim, Y.J.; Lee, J.H.; Jin, S.; Kim, J.H.; Kim, S.H. Primate-specific miR-944 activates p53-dependent tumor suppression in human colorectal cancers. *Cancer Lett.* **2019**, *440–441*, 168–179, doi:10.1016/j.canlet.2018.10.029.
152. Han, B.; Wang, X.; Yin, X. Knockdown of circRAD23B Exerts Antitumor Response in Colorectal Cancer via the Regulation of miR-1205/TRIM44 axis. *Dig. Dis. Sci.* **2021**, doi:10.1007/s10620-021-06859-w.
153. Guo, Y.; Zhu, Q.; Chen, S.; Li, Y.; Fu, D.; Qiao, D.; Ni, C. Post-transcriptional suppression of G protein-coupled receptor 15 (GPR15) by microRNA-1225 inhibits proliferation, migration, and invasion of human colorectal cancer cells. *3 Biotech* **2021**, *11*, doi:10.1007/s13205-021-02682-2.
154. Lin, W.; Zou, H.; Mo, J.; Jin, C.; Jiang, H.; Yu, C.; Jiang, Z.; Yang, Y.; He, B.; Wang, K. Micro1278 Leads to Tumor Growth Arrest, Enhanced Sensitivity to Oxaliplatin and Vitamin D and Inhibits Metastasis via KIF5B, CYP24A1, and BTG2, Respectively. *Front. Oncol.* **2021**, *11*, doi:10.3389/fonc.2021.637878.
155. Yang, F.; Wang, H.; Yan, B.; Li, T.; Min, L.; Chen, E.; Yang, J. Decreased level of miR-1301 promotes colorectal cancer progression via activation of STAT3 pathway. *Biol. Chem.* **2020**, doi:10.1515/hsz-2020-0301.
156. Chang, N.; Cui, Y.; Liang, X.; Han, D.; Zheng, X.; Wu, A.; Qian, L. Long Noncoding RNA LINC00857 Promotes Proliferation, Migration, and Invasion of Colorectal Cancer Cell through miR-1306/Vimentin Axis. *Comput. Math. Methods Med.* **2021**, *2021*, 1–9, doi:10.1155/2021/5525763.
157. Yang, D.; Li, R.; Xia, J.; Li, W.; Zhou, H. MiR-3666 suppresses cellular proliferation and invasion in colorectal cancer by targeting SATB2. *Mol. Med. Rep.* **2018**, *18*, 4847–4854, doi:10.3892/mmr.2018.9540.
158. Huang, L.; Zhang, Y.; Li, Z.; Zhao, X.; Xi, Z.; Chen, H.; Shi, H.; Xin, T.; Shen, R.; Wang, T. MiR-4319 suppresses colorectal cancer progression by targeting ABTB1. *United Eur. Gastroenterol. J.* **2019**, *7*, 517–528, doi:10.1177/2050640619837440.
159. Li, H.; Zhu, G.; Xing, Y.; Zhu, Y.; Piao, D. miR-4324 functions as a tumor suppressor in colorectal cancer by targeting HOXB2. *J. Int. Med. Res.* **2019**, *48*, doi:10.1177/0300060519883731.
160. Li, H.; Liang, W.; Zhang, H.; Shui, Y.; Zhang, Z. MicroRNA-4429 restrains colorectal cancer cell invasion and migration via regulating SMAD3-induced epithelial–mesenchymal transition. *J. Cell. Physiol.* **2021**, doi:10.1002/jcp.30271.
161. Li, H.; Liang, J.; Wang, J.; Han, J.; Li, S.; Huang, K.; Liu, C. Mex3a promotes oncogenesis through the RAP1/MAPK signaling pathway in colorectal cancer and is inhibited by hsa-miR-6887-3p. *Cancer Commun.* **2021**, doi:10.1002/cac2.12149
